# Genome-wide genetic variation and molecular surveillance of drug resistance in *Plasmodium falciparum* isolates from asymptomatic individuals in Ouélessébougou, Mali

**DOI:** 10.1038/s41598-023-36002-w

**Published:** 2023-06-12

**Authors:** Leen N. Vanheer, Almahamoudou Mahamar, Emilia Manko, Sidi M. Niambele, Koualy Sanogo, Ahamadou Youssouf, Adama Dembele, Makonon Diallo, Seydina O. Maguiraga, Jody Phelan, Ashley Osborne, Anton Spadar, Merel J. Smit, Teun Bousema, Chris Drakeley, Taane G. Clark, William Stone, Alassane Dicko, Susana Campino

**Affiliations:** 1 Department of Infection Biology, Faculty of Infectious and Tropical Diseases, London School of Hygiene and Tropical Medicine, London, UK; 2 Malaria Research and Training Centre, Faculty of Pharmacy and Faculty of Medicine and Dentistry, University of Sciences Techniques and Technologies of Bamako, Bamako, Mali; 3 Department of Medical Microbiology and Radboud Center for Infectious Diseases, Radboud University Medical Center, Nijmegen, The Netherlands; 4 Faculty of Epidemiology and Population Health, London School of Hygiene and Tropical Medicine, London, UK

**Keywords:** Parasite genetics, Parasite genomics, Antimicrobial resistance

## Abstract

Sequence analysis of *Plasmodium falciparum* parasites is informative in ensuring sustained success of malaria control programmes. Whole-genome sequencing technologies provide insights into the epidemiology and genome-wide variation of *P. falciparum* populations and can characterise geographical as well as temporal changes. This is particularly important to monitor the emergence and spread of drug resistant *P. falciparum* parasites which is threatening malaria control programmes world-wide. Here, we provide a detailed characterisation of genome-wide genetic variation and drug resistance profiles in asymptomatic individuals in South-Western Mali, where malaria transmission is intense and seasonal, and case numbers have recently increased. Samples collected from Ouélessébougou, Mali (2019–2020; n = 87) were sequenced and placed in the context of older Malian (2007–2017; n = 876) and African-wide (n = 711) *P. falciparum* isolates. Our analysis revealed high multiclonality and low relatedness between isolates, in addition to increased frequencies of molecular markers for sulfadoxine-pyrimethamine and lumefantrine resistance, compared to older Malian isolates. Furthermore, 21 genes under selective pressure were identified, including a transmission-blocking vaccine candidate (*pfCelTOS*) and an erythrocyte invasion locus (*pfdblmsp2)*. Overall, our work provides the most recent assessment of *P. falciparum* genetic diversity in Mali, a country with the second highest burden of malaria in West Africa, thereby informing malaria control activities.

## Introduction

Malaria was estimated to cause 250 million illnesses worldwide and 619 thousand associated deaths in 2021 alone. Mali is amongst the 9 countries with the highest burden of disease for malaria and its number of malaria cases has increased between 2016 and 2021^1^. Most malaria cases in Mali and in the rest of sub-Saharan Africa are caused by *Plasmodium falciparum,* the most virulent human malaria parasite. Mali is divided into five ecoclimatic zones^2^ across which malaria transmission fluctuates. The South-Western zone has the highest *P. falciparum* incidence rates and transmission is highly seasonal, coinciding with the annual rainy season. However, across different regions in South-Western Mali, the degree of perennial and seasonal transmission varies, as does the timing of the rainy season, causing heterogeneity in transmission seasons and malaria epidemiology^2^.

In accordance with WHO guidelines, current malaria intervention strategies in Mali include artemisinin combination therapies (ACTs) for uncomplicated *P. falciparum* malaria, with Artemether-Lumefantrine (AL) as the first-line treatment. Seasonal malaria chemoprevention (SMC) strategies, consisting of sulfadoxine-pyrimethamine with amodiaquine (SPAQ) in children and intermittent preventative treatment for pregnant women (IPTp) with sulfadoxine-pyrimethamine (SP) alone, have been widely implemented in African countries and have been introduced in Mali from 2012 and 2015 onwards, respectively.

In addition to disruption due to the COVID-19 pandemic, increased disease incidence has been linked to the emergence and spread of drug resistant *P. falciparum* parasites^1^. Drug-resistant strains against SP, artemisinin derivatives and partner drugs pose a major challenge in the fight against malaria^3^. Molecular markers of drug resistance are therefore extremely useful in identifying and monitoring drug-resistant *P. falciparum* parasites and have been described for most antimalarial drugs. Mutations in the multidrug resistance (*pfmdr1*) gene for example have been associated with various parasite responses to lumefantrine, chloroquine (CQ), amodiaquine, mefloquine and piperaquine. The genetic basis of SP resistance is well documented and involves an accumulation of mutations in the dihydrofolate-reductase (*pfdhfr*) gene (N51I, C59R, S108N and I164L) and the dihydropteroate-synthase (*pfdhps*) gene (S436A/F, A437G, K540E, A581G and A613S/T). Infections harbouring the triple *dhfr* C**IRN**I mutant (mutations underlined) are common throughout Africa and are pyrimethamine resistant^4,5^. The combination of this triple *dhfr* mutant with the double-mutant dhps (A437G and K540E, S**GE**AA) further increases the risk of SP treatment failure to 50%^5,6^.

Mutations in the *pfkelch13* gene associated with decreased artemisinin susceptibility emerged and spread in South-East Asia and have more recently emerged in several countries in East-Africa^7–9^. It is expected that this will spread to other parts of Africa and therefore continuous monitoring of *pfkelch13* genetic variation is critical. A high prevalence of CQ resistance led to its removal from any treatment guidelines for *P. falciparum* infections in sub-Saharan Africa. Decades after this, CQ-sensitive *P. falciparum* parasites have re-emerged in many parts of the world^10–13^. For that reason, a re-introduction of CQ, in combination with other antimalarials, has been proposed^14^. Reports from Mali have not observed a substantial decrease in frequency of mutations in the chloroquine resistance transporter (*pfcrt*) associated with CQ resistance^15^, although recently a downwards trend was reported in Malian isolates collected in 2016–2017^16^. Continued assessment is therefore important to determine whether CQ could be reintroduced in the region in future.

The majority of malaria infections in endemic areas are asymptomatic^17^, however, such carriers tend to be underrepresented in genome-wide large-scale genetic analyses, due to both the lack of seeking treatment and technical difficulties with sequencing low density infections. Asymptomatic carriers are the main contributors to the infectious reservoir as they can remain infectious for long periods of time without showing any symptoms, meaning they could unknowingly spread the disease to others while remaining unaware they are infected. In addition to this, their frequency in the population and the characteristics of individuals that are more likely to be asymptomatic, such as a higher risk of mosquito bites, further increase their contribution to the infectious reservoir^18,19^. Therefore, understanding the genetic characteristics of the asymptomatic reservoir is key to effectively controlling the spread of malaria.

Advances in Next-Generation Sequencing (NGS) technologies have rendered Whole Genome Sequencing (WGS) more accessible and affordable for use in disease management and malaria control. Along with identifying and monitoring molecular markers of drug resistance, investigating genomic variation is useful for understanding transmission dynamics, selective sweeps, and *P. falciparum* epidemiology. Assessing changes in genomic relatedness within a population, including through using identity-by-descent measures, can provide insights into parasite population demography and transmission intensity over time^20^. In addition, the identification of genes under selection can offer insights into the selective pressure exhibited by drugs or other unknown agents, which is important for developing effective strategies for prevention, control, and treatment of malaria.

In summary, monitoring of *P. falciparum* drug resistance is essential to inform drug policies worldwide, particularly in regions of high malaria transmission, and the WHO recommends regular updating and monitoring of antimalarial resistance to support progress made towards malaria control and elimination^21^. In this report, we provide an in-depth analysis of drug resistance profiles and recent genomic variation, using selection, ancestry and identity-by-descent analysis, in asymptomatic individuals in Ouélessébougou and neighbouring villages, Mali, in 2019 and 2020. Furthermore, we place these in the context of Malian *P. falciparum* isolates collected between 2007 and 2017, as well as African-wide parasite populations.

## Results

### Genomic population structure of Malian *P. falciparum* populations

**Figure 1:**
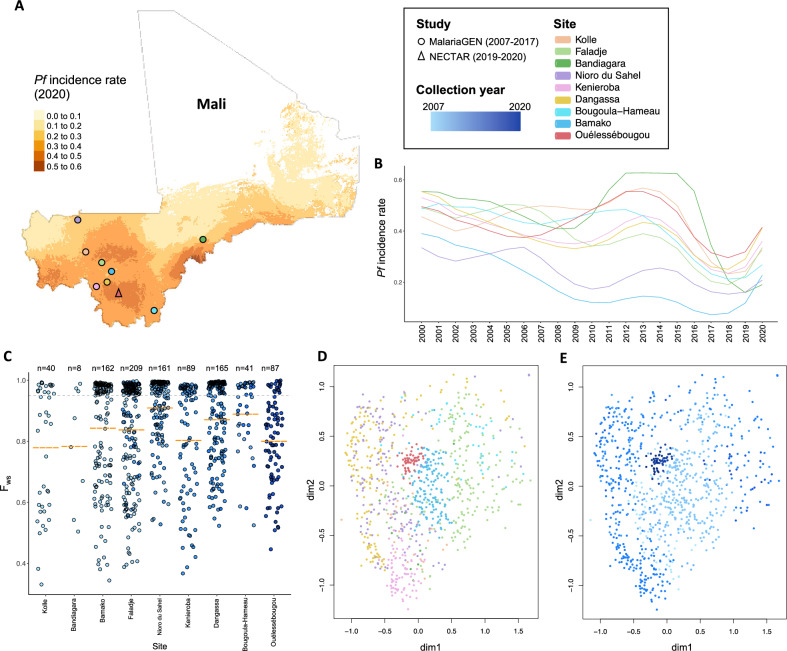
Population structure of *P. falciparum* isolates collected in Mali from 2007 to 2020. **(A)** Map of Mali presenting the number of newly diagnosed *Plasmodium falciparum* cases per 1,000 population in 2020 (colour scale) and indicating collection sites of the MalariaGEN studies (circle) and the New Drug Combinations for *P. falciparum* Transmission Reduction (NECTAR) clinical trials (triangle), coloured by village or city name. This map was generated using the *tmap* R package (version 3.3.3; https://r-tmap.github.io/tmap/) **(B)** *P. falciparum* incidence rate for each collection site (by colour) from 2000 to 2020. **(C)** Complexity of infections estimated by the in-breeding coefficient (F_ws_ metric) per sample, classified per study site, with the colour indicating collection year. Dotted grey trendline is at 0.95, dashed orange marks indicate mean F_ws_ value per group. **(D, E)** Uniform Manifold Approximation and Projection (UMAP) visualisation of 962 Malian *P. falciparum* isolates, coloured by site and collection year, respectively.

Genome-wide SNP analysis of 962 Malian *P. falciparum* isolates*,* collected between 2007 and 2020 in two different studies and originating in 9 locations (Fig. 1A), revealed differences in multiplicity of infection and population structure. Ouélessébougou isolates collected in 2019–2020 originated from asymptomatic *P. falciparum* infections, while no details on clinical presentation were available for publicly available genomes from the MalariaGEN database. *P. falciparum* incidence rates varied across sample sites and across two decades (Fig. 1A,B). A total of 863,046 high-quality SNPs were identified. Multiclonality was measured using the F_ws_ metric, or in-breeding coefficient, which is indicative of monoclonality if > 0.95, while a lower F_ws_ metric reflects multiclonality. The mean F_ws_ value for the 2019–2020 Mali samples (n = 87) from Ouélessébougou was 0.80, with only 20% of samples harbouring a single clone. We found a higher multiclonality in the 2019–2020 Ouélessébougou isolates compared to isolates from different sites where collection took place between 2015 and 2017 (Fig. 1C). Using the SNP data, a Uniform Manifold Approximation and Projection (UMAP) statistical analysis to cluster isolates revealed little spatial substructure between populations, although some grouping based on location (Fig. 1D) and collection year (Fig. 1E) could be observed and the Ouélessébougou isolates formed an individual subcluster.

### Genomic population structure of Malian *P. falciparum* populations in comparison to African-wide populations

**Figure 2:**
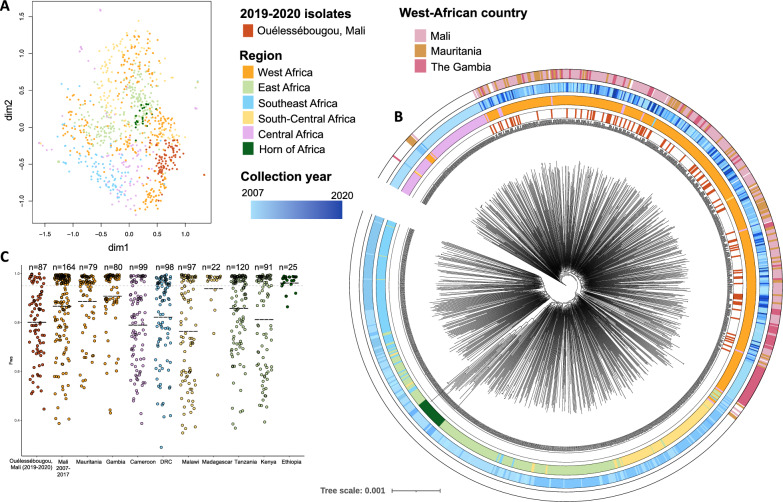
Population structure of Malian *P. falciparum* isolates in the context of African-wide populations. **(A)** Uniform Manifold Approximation and Projection (UMAP) visualisation of 962 African-wide *P. falciparum* isolates, including 87 isolates collected in Mali in 2019–2020 and 164 Malian isolates collected in 2007–2020. African regions where the isolates were collected are indicated by colour. **(B)** Maximum-Likelihood tree of the same dataset, annotated by the 2019–2020 Malian isolates (inner ring), the African region of sample origin (second ring), collection year (third ring) and West-African country of collection (outer ring). **(C)** Complexity of infections (F_ws_ coefficient) per sample, grouped by country and separating the 2019–2020 Malian isolates from the 2007 to 2017 Malian isolates. Dotted grey trendline is at 0.95, dashed lines indicate mean F_ws_ value per group.

A SNP-based UMAP visualisation and maximum likelihood tree show how the most recently collected Ouélessébougou isolates are related to African-wide populations (n = 875) and revealed similarity with West African and Central African isolates (Fig. 2A,B). No distinguishable clusters could be detected, indicating high relatedness, and suggesting movements of genetic information within these populations, which can occur both through human and vector migration. East African, Southeast African and Central African isolates formed separate clusters, while the Horn of Africa appeared to be a distinct cluster within the East African population. Multiclonality assessment showed that the 2019–2020 Malian isolates were relatively more multiclonal compared to the isolates collected in other West African countries (Fig. 2C).

### Frequencies of drug resistance molecular markers

**Figure 3:**
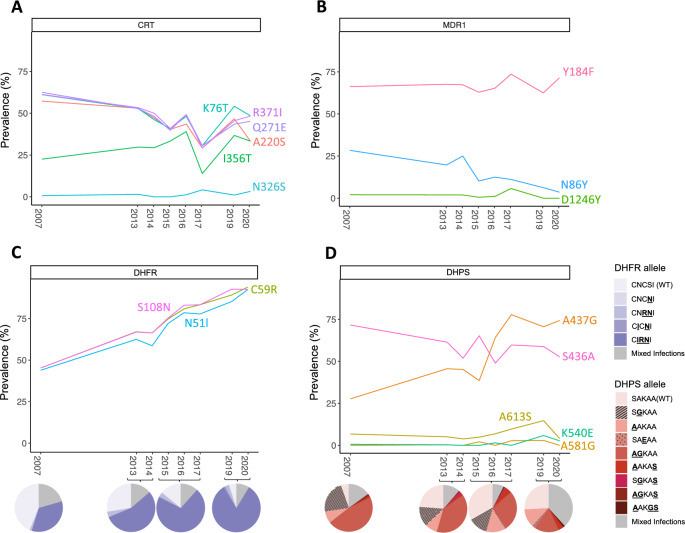
Prevalence of single nucleotide polymorphisms known to cause decreased drug susceptibility. Minor allele frequencies (MAFs) are shown for isolates collected from 2007 to 2020 in Mali for genes associated with drug resistance, including **(A)** *pfcrt*, **(B)** *pfmdr1*, **(C)** *pfdhfr* and **(D)** *pfdhps*. Pie charts in C and D represent the frequencies at which combinations of *pfhdfr* and *pfdhps* mutants were observed in the *P. falciparum* isolates collected in 2007, 2013–2014, 2015–2017 and 2019–2020.

Increased frequencies of molecular markers of sulfadoxine-pyrimethamine and persistence of chloroquine resistance markers were observed in the Ouélessébougou 2019–2020 population (n = 87), compared to Malian isolates from 2007 to 2017 (n = 876) (Table ST1). Polymorphisms causing amino acid changes that confer chloroquine resistance in *pfcrt* (K76T, A220S, Q271E, N326S, I356T and R371I) persisted at similar frequencies over time (Fig. 3A,B). The N86Y polymorphism on *pfmdr1* decreased over time from 28.4% in 2007 to 2.6% in 2020. This 86Y allele has been linked to chloroquine and amodiaquine resistance as well as piperaquine resistance (86Y allele in combination with Y184F), while parasites carrying the N86 allele show lower susceptibility to lumefantrine, piperaquine and mefloquine^22,23^. Y184F and D1246Y amino acid changes in MDR1 persisted at comparable frequencies. Three non-synonymous SNPs in *pfmdr1* that were not previously found in Malian isolates, resulting in amino acid changes S400C, D431Y and K503N, were identified in 2.13% of the 2019 isolates and 2.94% of the 2020 isolates, respectively (Table ST1). The frequency of point mutations in *pfdhfr* associated with pyrimethamine resistance (N51I, C59R and S108N) approximately doubled since 2007, reaching alarming frequencies of 92.4%, 93.9% and 92.7%, respectively, in 2020. In addition, the C**IRN**I triple mutant haplotype increased in frequency and made up 82.7% of the parasite population in 2019–2020, while the wild-type haplotype was reduced to 4.9% (Fig. 3C, Table ST2). One mutation in *pfdhps* conferring sulfadoxine resistance (A437G) increased in frequency from 27.7% in 2007 to 74.3% in 2020, while S436A showed a downwards trend (Fig. 3D). The *pfdhps* K540E mutation was found in 4 isolates in 2019–2020 and all of these were combined with the C**IRN**I triple *dhfr* mutant, leading to a quadruple mutant frequency of 2.47%. A non-synonymous SNP at position 748,145 in *pfdhfr* (V20I), was newly identified in Malian isolates in 2019, at 2.04% frequency. No known mutations in *pfkelch13* associated with artemisinin resistance were identified in any of the Malian isolates. The *pfkelch13* mutations R255K, K189N and K189T persisted at similar frequencies to the frequencies observed in 2007 and no new mutations in *pfkelch13* were identified (Table ST1).

### Regions under selective pressure in Malian isolates

**Figure 4:**
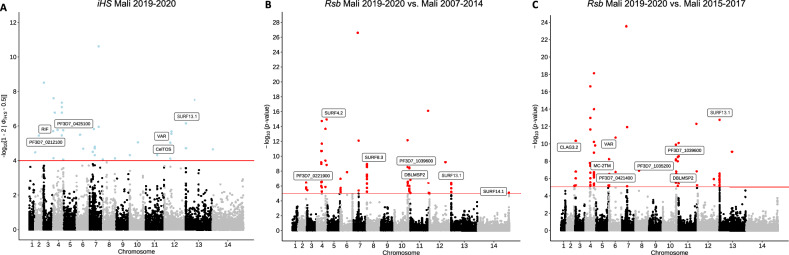
Scan for evidence of recent directional selection. Manhattan plots show analysis of the **(A)** integrated haplotype score (*iHS*) for individual SNPs in the 2019–2020 Mali population and *Rsb* cross-population test for extended haplotypes comparing the 2019–2020 Mali population to the **(B)** 2007–2014 Mali population and the **(C)** 2015–2017 Mali population.

Determination of genomic regions under directional selection by haplotype structure analysis within the Ouélessébougou 2019–2020 isolates and in comparison to the older Malian populations, revealed a number of genes to be under selective pressure. The integrated haplotype score (*iHS*) metric was used to identify SNPs under selection within the 2019–2020 population (Fig. 4A) and regions of the genome with an elevated number of SNPs under selection (Table ST3). This identified conserved *Plasmodium* protein coding genes with unknown function (PF3D7_0212100 and PF3D7_0425100), predominantly expressed in ookinetes and ring stages, respectively, as well as the *pfCelTOS* gene, which encodes a cell-traversal protein for ookinetes and sporozoites and was suggested as an attractive vaccine candidate antigen^24,25^. The between-population *Rsb* index was used to identify SNPs under selection when comparing the 2019–2020 population with the older Malian isolates collected in 2007–2014 (n = 414) (Fig. 4B) and 2015–2017 (n = 462) (Fig. 4C). Regions with a high number of SNPs under selection were determined as well (Table ST4). This identified genes associated with erythrocyte invasion (*pfdblmsp2, pfmsp3*), protein transport (*pfMC-2TM*), cytoadherence (*pfCLAG3.2*), and a gene encoding RNA of unknown function (Pf3D7_0421400, RUF6).

### Ancestral admixture analysis confirms similar ancestry among Malian isolates

**Figure 5:**
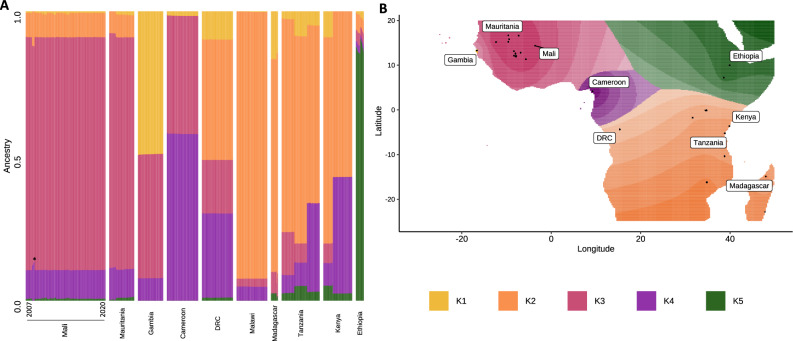
Genome-wide admixture ancestry proportions for *P. falciparum* populations across the African continent. **(A)** Ancestries per isolate (columns) for each country, ranked per country by ascending collection year. Asterisk indicates isolates from Bandiagara in Mali. **(B)** Geographic map of estimated ancestries using K = 5 ancestral populations across the African continent.

Spatial ancestry estimation of Malian isolates along with African-wide populations found similar ancestral origins among all Malian *P. falciparum* isolates. The optimum number of ancestral populations was estimated to be 5 (K = 5; K1–K5), based on eigenvalue decay corresponding to K ranging from 1 to 10. The K1 ancestral population was dominant in Gambian samples (49.4%), while the K2 ancestral population appeared to be linked to South-Central and East African populations (Malawi, 92.3%; Madagascar, 74.4%; Tanzania, 69%; Kenya, 61.9%). Malian isolates, along with Mauritanian isolates, seemed to contain mostly the K3 ancestral population (Mali, 80.3%; Mauritania, 80.5%), in addition to smaller portions of K2 ancestry (Mali, 8.5%; Mauritania, 7.7%) and K4 ancestry (Mali, 10%; Mauritania, 10.1%). Very low fractions of K1 and K5 ancestries were present in Malian and Mauritanian isolates, except for isolates from Bandiagara in Mali that appeared to not contain any K5 ancestry (Fig. 5).

### Identity-by-descent analysis reveals highly diverse Ouélessébougou population

As a measure of genetic relatedness within populations, identity-by-descent (IBD) analysis revealed that Ouélessébougou isolates exhibit very low fractions of pairwise IBD across the genome (median = 0, range = 0–0.133), while the Malian isolates collected in 2007–2014 and 2015–2017 showed a slightly higher relatedness (median = 0.021 and median = 0.017, respectively) (Fig. S2). The top 5% of IBD positions (classified in 10 kb windows of the genome) in the Ouélessébougou isolates were distributed across 17 regions on 3 chromosomes (chr. 6, 7 and 13) (Fig. S3, Table ST5). Three regions with high IBD on chromosome 6 included the gene encoding histone methyltransferase SET1 and the *pfcrt gene* on chromosome 7 was also identified as encompassing high IBD.

## Discussion

NGS technologies have provided an increasingly feasible method for exploring genome-wide genomic variation and population dynamics of malaria parasites. Here, we have provided a detailed analysis of genome-wide diversity of *P. falciparum* isolates from asymptomatic gametocyte carriers in 11 villages in Ouélessébougou in 2019 and 2020 and have placed them in the context of previously sequenced Malian isolates, as well as African-wide isolates via genome-wide SNP analysis. We found high multiclonality and low relatedness among isolates and identified genes under selective pressure. We also observed increased frequencies of molecular markers for sulfadoxine-pyrimethamine and lumefantrine resistance, compared to older Malian isolates.

Genomes from Malian *P. falciparum* isolates collected between 2007 and 2017 have previously been generated^16,26^. However, a more up-to-date and systematic sampling strategy is required to support efforts toward infection control and elimination. Ouélessébougou is a rural community in the Koulikoro region of South-Western Mali, which is an area with one of the highest *P. falciparum* incidence rates in the country^27^. Ideally, in such settings there should be regular monitoring of changes in genomic variation, to determine epidemiological patterns and population dynamics, especially among asymptomatic gametocyte carriers, and to assess the impact of infection control measures. For example, continued surveillance of drug resistance molecular markers is necessary to inform malaria chemotherapy approaches.

Malian isolates show minor clustering using UMAP visualisation, with Ouélessébougou isolates forming a subcluster. On a maximum-likelihood tree, Ouélessébougou and other Malian isolates cluster with West-African populations, as expected. Despite a recent report observing decreasing multiplicity of infection from 2007 to 2017 in Mali^16^, our findings show a higher multiclonality in 2019–2020 than in isolates from different sites where collection took place between 2015 and 2017. There may be multiple explanations for the highly multiclonal status of the study parasite population, such as the high *P. falciparum* incidence rates in this region (Fig. 1B), the timing of sample collection at peak transmission season (September-December), and the asymptomatic clinical presentation of the individuals. As asymptomatic *P. falciparum* gametocyte carriers are less likely to seek treatment, such infections may be prolonged, increasing the likelihood of reinfection and multiclonality. Despite most available sequencing data originating from incident infections, it is important to genetically characterise asymptomatic *P. falciparum* carriers, as these are the main contributors to the infectious reservoir and carry the infections that escape treatment^18^.

Malaria treatment strategies world-wide have been altered over time due to the emergence of resistant parasites to former first-line drugs, with the aim of preserving the efficacy of antimalarial drugs and reducing the global burden of malaria. We found that frequencies of molecular markers conferring CQ resistance have persisted in the 2019–2020 Malian isolates at similar frequencies compared to a decade ago, despite its removal from any *P. falciparum* treatment guidelines since 2006 and a report of a decreasing trend in 2017 in Mali^16^. This is unlike other areas in the world where a return in CQ sensitivity has been observed^11–14,28^ and could indicate a low fitness cost associated with maintaining *pfcrt* resistance polymorphisms in the population or a continued over the counter use of CQ^29–31^, thereby highlighting the need to investigate and reduce the availability of this drug in Mali and neighbouring countries.

We observed an increase in the N86 variant *pfmdr1* from 71.6% in 2007 to 97.4% in 2020. This N86 allele has been linked to lower susceptibility to lumefantrine, piperaquine and mefloquine^22,23^, while the 86Y allele previously showed chloroquine and amodiaquine resistance, as well as piperaquine resistance (86Y allele in combination with Y184F). As a result, AL was previously found to select for the N86 allele, whereas artesunate-amodiaquine and dihydroartemisinin-piperaquine select for the 86Y allele^32–35^. N86Y genotyping could therefore be a useful marker to guide rotation of ACTs in a given geographical area^36^. Thus, the observed increase in the N86 allele may reflect an expanding proportion of isolates with a decreased susceptibility to AL, thereby raising concern for a continued use of AL as the first-line ACT in Mali. A low frequency of the 86Y allele has been reported previously in Mali in 2016, as well as in other African countries where AL is widely used^15,34^. This finding is in contrast with a previously observed increase in the 86Y allele frequency in children in Ouélessébougou between 2014 and 2016, following 3 years of SMC with SPAQ, which was likely due to amodiaquine selecting for the 86Y allele^37^. However, it is important to note that, while a decreased susceptibility to AL in isolates with the N86 allele has been observed in isolates from multiple African countries^22,32,33^, this has not been phenotypically assessed in Malian isolates specifically.

High frequencies of triple *pfdhfr* C**IRN**I mutants (82.72%) were observed in the 2019–2020 Ouélessébougou isolates, which is a substantial increase from previous years (34.3%, 55.1% and 70.7% in 2007, 2013–2014 and 2015–2017, respectively). The *pfdhps* K540E mutant, which was quasi absent in the Malian isolates collected between 2007 and 2017, was found in 5.9% of 2019 isolates and 2.7% of 2020 isolates. In addition, all K540E mutant isolates harboured the triple *dhfr* mutant. Continued surveillance is therefore needed to monitor levels of SP resistance and the emergence of quadruple and quintuple (in combination with *dhps* A437G or *dhps* A581G) mutants. Moreover, online mutation prevalence maps such as wwarn.org SP molecular surveyor will become increasingly useful to assess which drug to use for SMC^38^. No SNPs at positions in the *pfkelch13* gene that have been reported to cause decreased susceptibility to artemisinin derivatives were observed in any Malian isolates, however, further monitoring for *pfkelch13* mutants over the next years is needed, as a spread of these alleles from East-Africa or their independent emergence can be expected.

Analysis of haplotype structure in the 2019–2020 Ouélessébougou isolates identified SNPs under selective pressure in the *pfCelTOS* gene, which is involved in sporozoite gliding motility, cell traversal, and is a transmission-blocking vaccine candidate^24,25^. Selective pressure has been observed at this genomic location before^39^, indicating a high degree of diversity and thereby rendering *pfCelTOS* a less attractive vaccine candidate. Cross-population analysis comparing parasite populations from 2019–2020 to 2007–2014 and 2015–2017 was performed and, despite increasing frequencies of molecular markers of drug resistance over this 13 year period, we did not find any drug resistance associated SNPs under directional selective pressure. We identified SNPs under selective pressure in genes associated with erythrocyte invasion (*pfdblmsp2)* in both population comparisons and genes associated with protein transport (*pfMC-2TM*), cytoadherence (*pfCLAG3.2*), and a gene encoding RNA of unknown function (Pf3D7_0421400, RUF6) in the comparison between the 2019–2020 and 2015–2017 populations. Selective pressure in *pfdblmsp2* and members of the clag multigene family has been described before and is likely due to their location as surface proteins and the resulting contact with the host immune system^39,40^. IBD analysis revealed very low fractions of pairwise IBD across the genome in isolates from 2019–2020 Ouélessébougou, indicating low relatedness between isolates. The *pfcrt* gene, associated with CQ and piperaquine resistance was found to be in the top 5% of IBD positions, suggesting that this gene is highly conserved among Ouélessébougou isolates. This is in accordance with the observed persistent frequencies of molecular markers for CQ resistance. In addition, the *pfSET1* gene, an import histone lysine methyltransferase, was highly conserved as well. Admixture ancestry analysis showed similar ancestries for all Malian isolates, which is in line with a recent report^16^.

This study had several limitations. Firstly, sampling bias cannot be excluded as we only sequenced parasites from asymptomatic infections and from individuals between the ages of 5 and 50, while publicly available datasets do not specify the clinical presentation or individual’s age. In addition, copy number analysis of *plasmepsin1-2*, which confers piperaquine resistance, was not performed, due to selective Whole Genome Amplification (sWGA) of parasite DNA prior to sequencing, which prevents adequate analysis of copy number. Lastly, we did not assess phenotypic resistance or any association between genotype and phenotype, however, the molecular markers for drug resistance reported here have been widely proven to predict either in vitro drug resistance levels or patient treatment outcomes^4–6,41–43^.

After years of progress towards reducing the global burden of malaria, incidence rates and deaths are now on the rise. This rise could be due to disruptions in malaria control programmes during the COVID-19 pandemic, but antimalarial resistance has been linked to this increase as well. In order to progress towards the goal of reducing the global malaria burden by 90% by 2030^44^, we need to generate a comprehensive picture of the genomic variation and the epidemiology of parasite populations. Here, we have provided an updated assessment of genomic diversity of the *P. falciparum* parasite population in South-Western Mali, a region with very intense malaria transmission. Our results showed that the parasites originating from Mali clustered according to their geographic and temporal origin, with the Ouélessébougou isolates forming a separate subcluster. This suggests a high genetic diversity among Malian isolates. Molecular markers of SP resistance were found to be on the rise, which shows a progression towards failure of this drug combination and necessitates continued monitoring. No decline in CQ resistance over time was observed, opposing the idea of a potential CQ re-introduction in Mali in the near future. Our study contributes valuable data regarding the current epidemiological and drug resistance scenario of malaria in Mali and can aid effective malaria control in Mali. Further applications of sequencing approaches, including new portable technologies and amplicon sequencing assays, in malaria endemic countries are needed to assist disease control and inform treatment guidelines.

## Methods

### Study sites

In 2019 and 2020 a total of 180 individuals with microscopy detectable *P. falciparum* gametocytes in the absence of malaria symptoms were recruited into two clinical trials^45,46^ in Ouélessébougou and 11 villages in Ouélessébougou, Mali (Fig. S1). Ouélessébougou is a commune that includes the town of Ouélessébougou and 44 surrounding villages, which have a total of approximately 50,000 inhabitants. The town is located about 80 km south from Bamako, the capital city of Mali. Malaria transmission in Ouélessébougou is highly seasonal occurring during rainy season from July to November. Publicly available WGS data from Malian isolates originated from an additional 8 locations across Southern Mali, largely consisting of rural villages (Bougoula-Hameau, Dangassa, Faladje, Kenieroba, Kolle, Nioro-du-Sahel, Bandiagara) and one urban area (Bamako). All sites have a subtropical climate with dry and rainy seasons, except Nioro-du-Sahel, which is characterised by a desert climate^2,16^.

### Sample collection and whole genome sequencing

A total of 97 whole blood samples were selected from *P. falciparum* gametocyte carriers, aged between 5 and 50 years, recruited into two previously published clinical trials in Ouélessébougou^45,46^. Permission to conduct this study was obtained from the London School of Hygiene and Tropical medicine Research Ethics Committee (reference numbers 17507 and 21905) and the University of Sciences Techniques and Technologies of Bamako Ethical Committee (reference numbers 2019/67/CE/FMPOS and 2020/96/CE/FMPOS/FAPH) and performed in accordance with relevant guidelines and regulations. The trials were registered on ClinicalTrials.gov (NCT04049916 and NCT04609098). Written informed consent was obtained from all subjects and/or their legal guardians prior to sample collection. For minor participants, informed consent for study participation was obtained from their parent and/or legal guardian. Species identification was carried out by microscopy by trained microscopists at the Malaria Research and Training Centre of the University of Bamako (Bamako, Mali). DNA was extracted from 83.3 μL whole blood using a MagNAPure LC automated extractor (Total Nucleic Acid Isolation Kit High Performance; Roche Applied Science, Indianapolis, IN, USA) and amplified using an established selective whole genome amplification (sWGA) primer set and protocol^47,48^. Whole genome sequencing was performed on an Illumina Novaseq 6000 platform at Eurofins Genomics, Germany, rendering a minimum of 3.75 M paired reads (250 bp reads) per sample.

### Data set selection, read mapping, variant detection and quality control

A total of 1701 *P. falciparum* isolates were included in the analysis (Supplementary File S2), including publicly available whole genome sequences Malaria Genetic Epidemiology Network^26^ (MalariaGEN) (Pf Community Project, n = 1141; SPOTmalaria project, n = 463) and newly sequenced isolates (n = 97) from asymptomatic *P. falciparum* infected individuals recruited into two previously published clinical trials in Ouélessébougou, Mali^45,46^. All raw sequence data was filtered using *trimmomatic* (version 0.39) and the following parameters: LEADING:3 TRAILING:3 SLIDINGWINDOW:4:20 MINLEN:36. Illumina reads were then mapped to the *P. falciparum* (Pf3D7; v3) reference genome using *bwa-mem* software (v0.7.17). SNPs and short insertions and deletions were called using *samtools* (v1.12) and GATK (v4.1.4.1) software. Mixed call SNPs were assigned genotypes determined by a ratio of coverage in which nucleotide calls were 80% or higher. Samples with more than 40% missingness were not included in any analysis. Of the 97 newly sequenced samples, 9 were removed due to missingness and one was removed after species prediction identified *P. malariae* (https://github.com/jodyphelan/malaria-profiler), leaving a total of 87 (89.6%) newly sequenced isolates in the final analyses. Of the publicly available datasets, 8 were removed, resulting a total of 1673 samples included in the analyses, including isolates from Cameroon (n = 99), Democratic Republic of Congo (DRC; n = 98), Gambia (n = 80), Kenya (n = 91), Malawi (n = 97), Mali (n = 962), Mauritania (n = 79), Tanzania (n = 120), Madagascar (n = 22) and Ethiopia (n = 25).

### Population genetic analyses

Visualisation of sample site geography was performed using the *ggmap* (version 3.0.0) and *tmap* (version 3.3.3) R packages. *P. falciparum* incidence rates from 2000 to 2020 were accessed from the Malaria Atlas Project^27^. Uniform Manifold Approximation and Projection (UMAP) plots were created using the *uwot* R package with ‘hamming’ metric and default parameters. A maximum likelihood tree was created by applying *iqtree* software using genome-wide SNPs, and visualisation was performed in *iTOL* (version 6)^49,50^. For population genetic analyses that compare Malian isolates to African-wide populations, a subset of Malian isolates collected between 2007 and 2017 was used (including 10 isolates from each site and each collection year, if available), to obtain comparable number of isolates between populations. Multiclonality was determined by calculating the F_ws_ metric, using an in-house script that utilizes the moimix R package (https://github.com/bahlolab/moimix) to assess within-host diversity in relation to the local population diversity. Only bi-allelic SNPs in coding regions were used for the calculations and Minor Allele frequencies (MAFs) filtering of 0.1% was performed in order to exclusively include robust SNPs. MAFs in drug resistance genes were extracted from the binary matrix and annotated using *Bcftools CSQ* software, which identifies the mutation as non-synonymous, synonymous, or intergenic as well as the codon and protein shift caused by any non-synonymous mutations^51^. Only genomic positions with MAFs of at least 2% were retained in the analysis. Data visualisation was performed using the R-based *ggplot2* package (R version 4.1.2). Regions of the genome under directional selection were identified using the *rehh* R package (version 3.2.2), which uses population-based measures of haplotype diversity within (*iHS*) or between (*Rsb*) populations^52^. The R-based *Tess3r* package (version 1.1.0, using default parameters apart from ‘rep = 25’). was used to calculate admixture based on the spatial modelling of allele sharing using geographical coordinates from sampling sites in addition to genome-wide SNP data^53^. The optimal number of ancestries was determined across different numbers of sub-populations (K = 1, 2,…, 10). IBD analysis for isolates with F_ws_ > 0.85 was performed to assess connectivity between parasites within populations. This was achieved by estimating the pairwise fraction of shared ancestry between genomic segments, which were inferred to have descended from a recent common ancestor. These IBD fractions were calculated using the *hmmIBD* software with default parameters, which deploys a hidden Markov model-based approach^54^. French translation of the manuscript is available in Supplementary File S3 and was assisted by DeepL Translator (https://www.deepl.com/translator).

## Supplementary Information

Supplementary Information 1.

Supplementary Information 2.

Supplementary Information 3.

## Data Availability

The datasets presented in this study can be found in European Nucleotide Archives (ENA). The names and sample accession number(s) can be found in the Supplementary File S1. Raw sequences for the isolates sequenced in this study are available from the ENA website (Project accession PRJEB60381).
