# Supplementary material for: Genome-wide genetic variation and molecular surveillance of drug resistance in Plasmodium falciparum isolates from asymptomatic individuals in Ouélessébougou, Mali
Source: Sci Rep. 2023 Jun 12;13:9522. doi: 10.1038/s41598-023-36002-w (PMC10258750; doi:10.1038/s41598-023-36002-w)
Supplement: Supplementary file 1 — Supplementary Information 1. [file 41598_2023_36002_MOESM1_ESM.pdf]

Genome-wide genetic variation and molecular surveillance of drug resistance in *Plasmodium falciparum* isolates from asymptomatic individuals in Ouélessébougou, Mali.

Leen N. Vanheer[1], Almahamoudou Mahamar[2], Emilia Manko[1], Sidi M. Niambele[2], Koualy Sanogo[2], Ahamadou Youssouf[2], Adama Dembele[2], Makonon Diallo[2], Seydina O. Maguiraga[2], Jody Phelan[1], Ashley Osborne[1], Anton Spadar[1], Merel J. Smit[3], Teun Bousema[3], Chris Drakeley[1], Taane G. Clark[1,4], William Stone[1], Alassane Dicko[2], Susana Campino[1]

[1] Faculty of Infectious and Tropical Diseases, London School of Hygiene & Tropical Medicine, London, UK

[2] Malaria Research and Training Centre, Faculty of Pharmacy and Faculty of Medicine and Dentistry, University of Sciences Techniques and Technologies of Bamako, Bamako, Mali

[3] Department of Medical Microbiology and Radboud Center for Infectious Diseases, Radboud University Medical Center, Nijmegen, Netherlands

[4] Faculty of Epidemiology and Population Health, London School of Hygiene & Tropical Medicine, London, UK

Corresponding authors

Dr. Leen Vanheer and Prof. Susana Campino

Department of Infection Biology

London School of Hygiene & Tropical Medicine,

London WC1E 7HT, UK

leen.vanheer@lshtm.ac.uk, susana.campino@lshtm.ac.uk

SUPPLEMENTAL FIGURES

Supplemental Figure S1. Sampling sites in Ouélessébougou. Location of 11 sample sites (red dots) in Ouélessébougou and surrounding villages where *P. falciparum* blood samples were collected in 2019 and 2020. This map was generated using the *ggmap* R package (version 3.0.0; https://github.com/dkahle/ggmap).

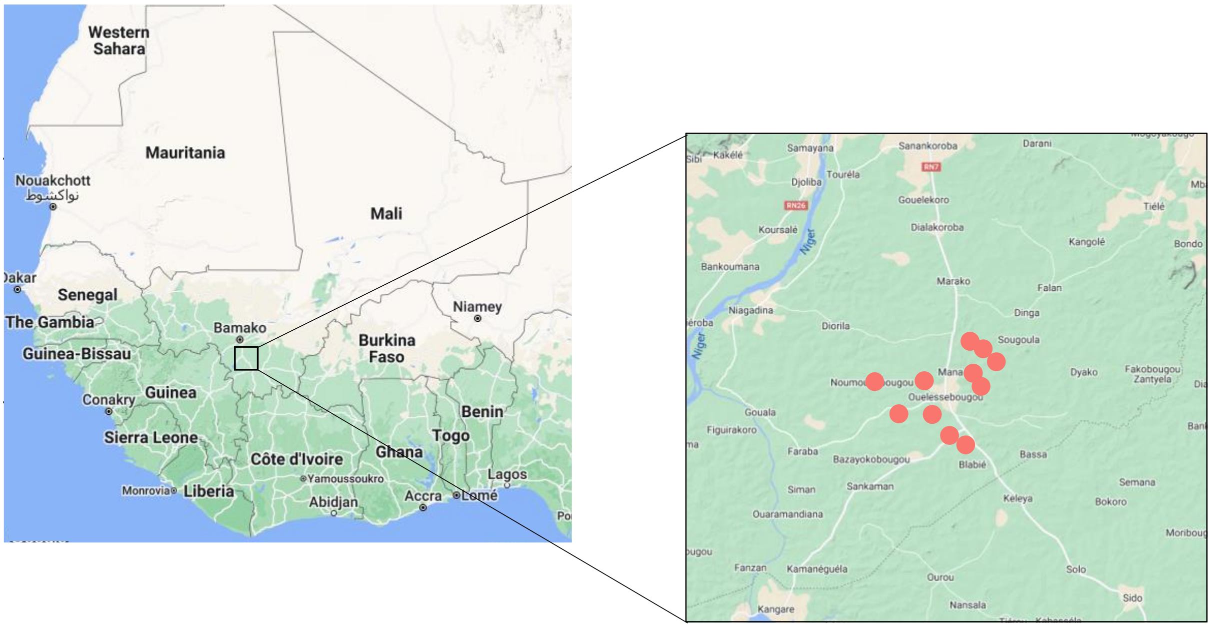

Supplemental Figure S2. Pairwise Identity-by-descent (IBD)-fractions across country-level populations of *P. falciparum* isolates. Isolates with F_{ws}>0.85 were incorporated in the analysis and include isolates from Mali 2007-2014 (67), Mali 2015-2017 (97), Mali 2019-2020 (87), Mauritania (79), Gambia (80), Cameroon (99), DRC (98), Malawi (97), Madagascar (22), Tanzania (120), Kenya (91) and Ethiopia (25) populations. All boxplots consist of boxes (median and interquartile range) and whiskers that extend to the most extreme data point which is no more than 1.5 times the interquartile range from the box.

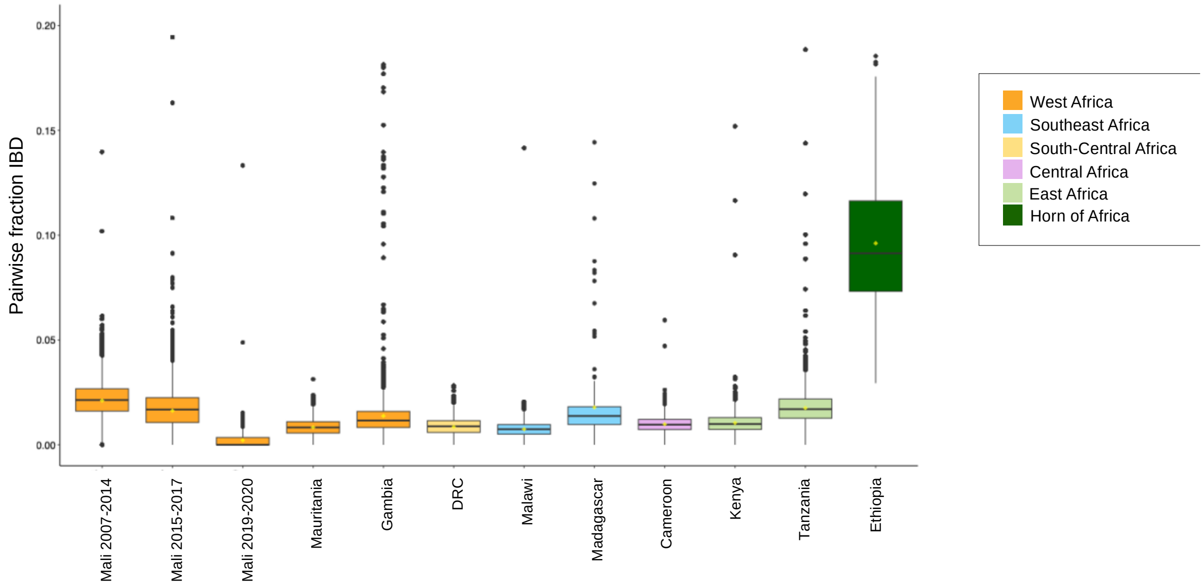

Supplemental Figure S3. Genome-wide distribution of pairwise identity-by-descent (IBD) fractions across country-level populations of *P. falciparum* isolates. Isolates with F_{ws}>0.85 were incorporated in the analysis and include isolates from Mali 2007-2014 (67), Mali 2015-2017 (97), Mali 2019-2020 (87), Mauritania (79), Gambia (80), Cameroon (99), DRC (98), Malawi (97), Madagascar (22), Tanzania (120), Kenya (91) and Ethiopia (25) populations. Top 5% of genomic regions for the Mali 2019-2020 population are presented in Supplemental Table S4.

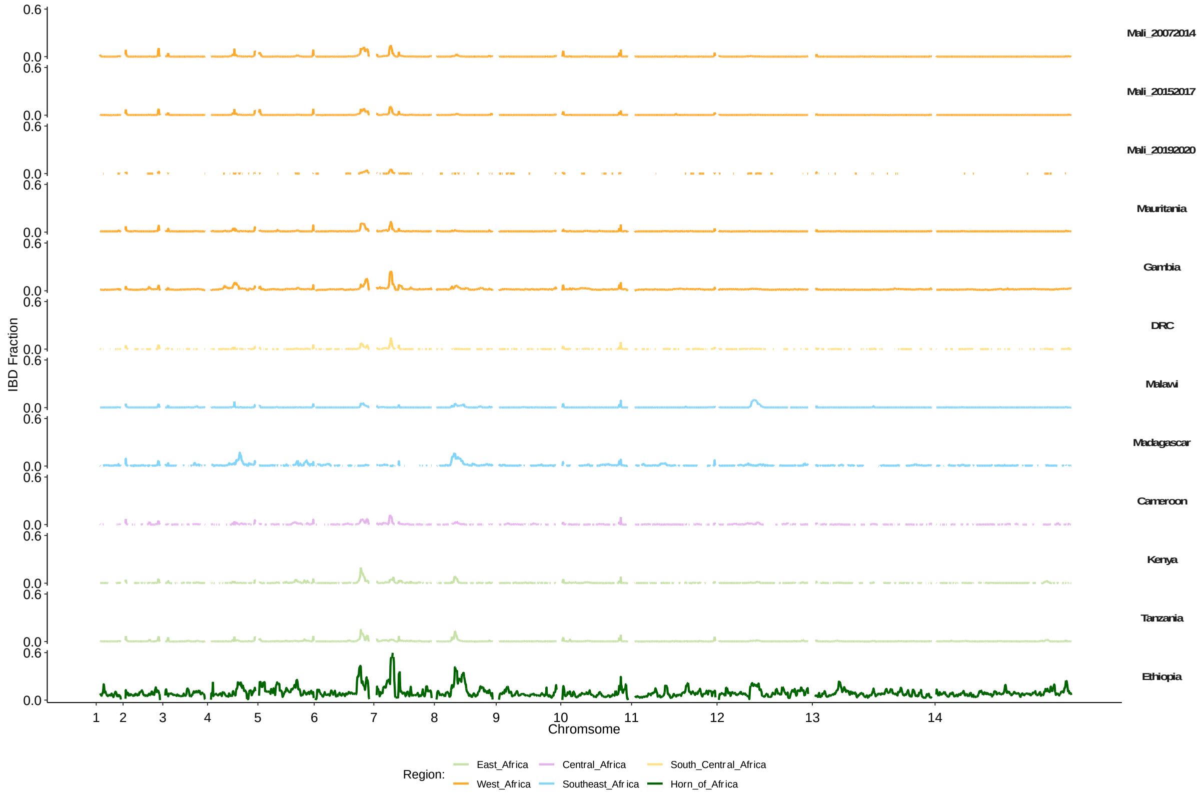

SUPPLEMENTAL TABLES

Supplemental Table ST1. Non-synonymous single nucleotide polymorphisms in genes associated with drug resistance. Positions with minor allele frequency (MAF) of at least 2% were retained in this table. Frequencies are presented for each sampling year in Mali.

Gene	Gene ID	Chromosome	Nt change	AA change	MAF 2007 (%) n=74	MAF 2012 (%) n=4	MAF 2013 (%) n=284	MAF 2014 (%) n=52	MAF 2015 (%) n=84	MAF 2016 (%) n=342	MAF 2017 (%) n=36	MAF 2019 (%) n=51	MAF 2020 (%) n=36
MDR1	PF3D7_0523000	5	957990A>G	K34R	0.00	33.33	1.06	0.00	1.20	0.44	1.39	2.17	0.00
			958145A>T	N86Y	28.38	0.00	19.72	25.00	11.31	12.17	11.11	6.25	3.57
			958440A>T	Y184F	66.22	66.67	67.61	67.31	66.27	64.56	72.22	62.50	71.43
			958496C>T	P203S	0.00	0.00	0.53	0.00	1.20	0.29	2.86	1.06	0.00
			959087A>T	S400C	0.00	0.00	0.00	0.00	0.00	0.00	0.00	2.13	0.00
			959180G>T	D431Y	0.00	0.00	0.00	0.00	0.00	0.00	0.00	2.13	0.00
			959398A>T	K503N	0.00	0.00	0.00	0.00	0.00	0.00	0.00	0.00	2.94
			959401T>A	N504K	4.05	0.00	4.05	5.77	1.22	4.33	2.78	3.33	4.41
			960702T>A	F938Y	0.00	0.00	4.05	5.77	0.62	1.55	7.14	3.00	1.56
			961462G>A	M1191I	0.00	0.00	0.35	0.00	0.00	0.30	0.00	3.06	0.00
			961473A>C	Q1195P	0.00	0.00	0.18	0.00	0.00	0.00	2.78	0.00	0.00
			961494A>G	N1202S	0.00	0.00	0.00	0.00	0.00	0.30	0.00	2.00	0.00
			961625G>T	D1246Y	2.03	0.00	1.94	1.92	1.90	0.74	5.71	0.00	0.00
CRT	PF3D7_0709000	7	403625A>C	K76T	61.11	100.00	52.82	46.15	36.31	48.98	31.94	54.17	48.48
			404407G>T	A220S	57.26	100.00	53.01	47.12	36.31	44.41	30.56	46.67	33.33
			404836C>G	Q271E	62.50	100.00	52.82	48.08	36.31	49.71	31.94	43.48	45.16
			404948A>T	Y276F	0.00	0.00	0.00	0.00	0.00	0.00	2.78	0.00	0.00
			405362A>G	N326S	0.74	0.00	1.44	0.00	0.60	1.03	4.17	1.04	3.23
			405600T>C	I356T	22.54	100.00	29.75	29.41	28.57	39.91	15.28	36.73	33.33
			405838G>T	R371I	61.27	100.00	53.35	50.00	36.90	49.85	30.56	45.56	48.28

K13	PF3D7_1343700	13	1726226A>T	L258M	1.35	0.00	1.06	0.00	0.00	0.44	0.00	2.00	0.00
			1726234C>T	R255K	4.05	16.67	2.29	0.00	3.61	2.51	0.00	0.00	1.43
			1726349T>G	N217H	0.00	0.00	0.00	0.00	0.00	0.00	2.78	0.00	0.00
			1726431T>A	K189N	6.76	0.00	4.40	2.88	3.05	3.54	11.43	7.84	6.76
			1726432T>G	K189T	52.70	83.33	49.82	62.50	44.23	54.91	44.29	50.00	56.76
			1726663C>T	G112E	0.00	0.00	1.76	0.00	5.95	1.32	0.00	0.00	0.00
			1726697T>C	R101G	0.00	0.00	0.18	0.00	0.60	0.44	2.78	0.00	0.00
			1726737G>C	N87K	0.00	0.00	0.00	0.00	0.00	0.00	2.78	1.96	0.00
DHFR	PF3D7_0417200	4	748145G>A	V20I	0.00	0.00	0.00	0.00	0.00	0.00	0.00	2.04	0.00
			748239A>T	N51I	43.92	83.33	62.50	58.65	82.74	75.88	76.39	85.29	92.42
			748262T>C	C59R	45.27	83.33	67.08	66.35	83.93	78.59	81.94	89.22	93.94
			748410G>A	S108N	45.27	83.33	66.90	66.35	83.33	80.99	81.94	92.71	92.65
			748558T>G	N157K	0.00	0.00	0.00	0.00	0.00	0.00	2.78	0.00	0.00
			748854A>G	K256R	0.00	0.00	0.00	2.88	0.00	0.00	0.00	0.00	0.00
DHPS	PF3D7_0810800	8	548940G>C	E189Q	2.03	0.00	0.53	0.00	0.00	1.46	6.94	0.00	0.00
			549666A>G	I431V	0.00	0.00	0.53	0.00	1.19	0.29	2.78	3.92	0.00
			549681T>G	S436A	71.62	50.00	61.44	51.92	60.12	50.44	61.11	58.82	52.70
			549685G>C	A437G	27.70	100.00	45.60	45.19	38.60	61.76	76.39	70.59	74.32
			549993A>G	K540E	0.68	0.00	0.35	0.00	0.00	1.47	0.00	5.88	2.70
			550117C>G	A581G	0.00	0.00	0.35	0.00	1.19	0.29	2.78	2.94	0.00
			550212G>T	A613S	6.76	0.00	5.11	3.85	4.17	7.04	9.72	14.71	4.05

Nt = Nucleotide; AA = Amino Acid

Supplemental Table ST2. Observed frequencies of *dhfr* and *dhps* haplotypes. Frequencies of combinations of SNPs in *dhfr* and *dhps* known to confer SP resistance are reported. Malian isolates are grouped by collection year.

Gene	Haplotype	Frequency 2007 (%) n=74	Frequency 2013-2014 (%) n=336	Frequency 2015-2017 (%) n=462	Frequency 2019-2020 (%) n=87
DHFR	CNCSI	43.84	27.68	13.97	4.94
	CIRNI	34.25	55.06	70.74	82.72
	CICNI	0.00	0.00	0.66	1.23
	CNRNI	1.37	3.57	2.84	2.47
	CNCNI	0.00	0.00	0.22	0.00
	Mixed Infections	20.55	13.69	11.57	8.64
DHPS	SAKAA	5.48	23.81	32.97	25.93
	AAKAA	8.22	8.63	12.88	12.35
	SGKAA	10.96	6.55	6.33	0.00
	SAEAA	0.00	0.00	0.87	2.47
	AGKAA	46.58	39.29	27.07	16.05
	AAKAS	1.37	1.49	4.59	3.70
	SGKAA	10.96	6.55	6.33	0.00
	SGKAS	0.00	0.89	0.44	0.00
	AGKAS	1.37	0.30	0.00	0.00
	SGKAS	0.00	0.89	0.44	0.00
	AAKGS	0.00	0.30	0.66	1.23
	Mixed Infections	15.07	11.31	7.42	38.27
DHFR+DHPS	CIRNI+SAEAA	0.00	0.00	0.66	2.47

Supplemental Table ST3. Candidate regions in positive selection analysis using the integrated haplotype score (*iHS*) in Ouélességbougou (Mali 2019-2020) population.

Chr	Start	End	Number of markers	Mean iHS markers	Max iHS	Number of iHS extreme markers (iHS p < 1x10-4, two-sided)	Percentage of extreme iHS markers (iHS p < 1x10-4, two-sided)	Mean iHS of extreme markers (iHS p < 1x10-4, two-sided)	Genes in the region	Products
4	110000	140000	93	0.648	7.899	3	3.23	5.571	PF3D7_0402100	Plasmodium exported protein (PHISTb), unknown function
7	590000	620000	166	0.638	5.175	4	2.41	4.489	PF3D7_0713600	ribosomal protein S5, mitochondrial, putative
12	640000	670000	22	1.085	5.628	2	9.09	5.075	PF3D7_1216700(PLP2)	perforin-like protein 2
13	90000	120000	96	0.838	6.023	2	2.08	5.597	PF3D7_1302000(PTP6); PF3D7_1301700(CBP2)	EMP1-trafficking protein; CX3CL1-binding protein 2
13	2490000	2520000	11	2.087	10.641	2	18.18	10.316	PF3D7_1362500(EXO); PF3D7_1362700; PF3D7_1362200(RUVB3)	3'-5' exonuclease, putative; conserved Plasmodium protein, unknown function; RuvB-like helicase 3

Supplemental Table ST4. Candidate regions in positive selection analysis using the between populations (*Rsb*) metric comparing the Ouélessébougou (Mali 2019-2020) population with the Mali 2007-2014 and Mali 2015-2017 populations.

Population comparison	Chr	Start	End	Number of markers	Mean Rsb markers	Max Rsb	Number of Rsb extreme markers	Percentage of extreme Rsb markers	Mean Rsb of extreme markers	Genes in the region	Products
Mali 2007-2014 vs Mali 2019-2020	2	860000	890000	66	0.962	6.486	3	4.55	5.954	PF3D7_0221800	hypothetical protein
Mali 2007-2014 vs Mali 2019-2020	4	530000	560000	54	1.566	10.727	8	14.81	8.08	PF3D7_0412100(mtRPS12)	ribosomal protein S12, mitochondrial
Mali 2007-2014 vs Mali 2019-2020	4	590000	620000	210	0.747	14.738	10	4.76	8.515	PF3D7_0413600(RPT3)	26S protease regulatory subunit 6B, putative
Mali 2007-2014 vs Mali 2019-2020	4	1090000	1120000	178	0.534	14.937	2	1.12	11.893	PF3D7_0424300(EBA165)	erythrocyte binding antigen-165, pseudogene
Mali 2007-2014 vs Mali 2019-2020	6	40000	80000	79	1.383	7.005	4	5.06	5.83	PF3D7_0601500	Plasmodium exported protein (PHISTb), unknown function
Mali 2007-2014 vs Mali 2019-2020	7	590000	620000	294	0.846	12.113	2	0.68	8.736	PF3D7_0713600	ribosomal protein S5, mitochondrial, putative
Mali 2007-2014 vs Mali 2019-2020	10	1380000	1410000	238	0.859	12.178	6	2.52	8.413	PF3D7_1035400(MSP3); PF3D7_1034900(MRScyt)	merozoite surface protein 3; methionine--tRNA ligase
Mali 2007-2014 vs Mali 2019-2020	10	1580000	1610000	23	2.564	8.49	7	30.43	6.37	PF3D7_1040200	stevor
Mali 2007-2014 vs Mali 2019-2020	13	90000	120000	122	1.243	6.454	4	3.28	5.869	PF3D7_1302000(PTP6); PF3D7_1301700(CBP2)	EMP1-trafficking protein; CX3CL1-binding protein 2

Mali 2015-2017 vs Mali 2019-2020	3	110000	140000	206	0.825	10.366	4	1.94	7.117	PF3D7_0302300; PF3D7_0302500(CLAG3.1); PF3D7_0302200(CLAG3.2)	erythrocyte membrane protein 1 (PfEMP1), pseudogene; cytoadherence linked asexual protein 3.1; cytoadherence linked asexual protein 3.2
Mali 2015-2017 vs Mali 2019-2020	4	530000	560000	56	1.019	7.812	2	3.57	7.812	PF3D7_0412100(mtRPS12)	ribosomal protein S12, mitochondrial
Mali 2015-2017 vs Mali 2019-2020	4	590000	620000	205	0.911	16.604	11	5.37	8.429	PF3D7_0413600(RPT3)	26S protease regulatory subunit 6B, putative
Mali 2015-2017 vs Mali 2019-2020	6	50000	80000	59	1.573	6.537	3	5.08	5.614	PF3D7_0601500	Plasmodium exported protein (PHISTb), unknown function
Mali 2015-2017 vs Mali 2019-2020	6	710000	740000	16	2.032	10.73	2	12.5	8.746	PF3D7_0617200); PF3D7_0617600; PF3D7_0617100	BFR1 domain-containing protein, putative; stevor; AP-2 complex subunit alpha, putative
Mali 2015-2017 vs Mali 2019-2020	10	1380000	1410000	239	0.683	9.906	6	2.51	7.723	PF3D7_1035400(MSP3); PF3D7_1034900(MRScyt)	merozoite surface protein 3; methionine--tRNA ligase
Mali 2015-2017 vs Mali 2019-2020	10	1580000	1610000	24	2.476	8.486	7	29.17	6.454	PF3D7_1040200	stevor
Mali 2015-2017 vs Mali 2019-2020	13	90000	120000	119	2.335	12.761	19	15.97	6.226	PF3D7_1302000(PTP6); PF3D7_1301700(CBP2)	EMP1-trafficking protein; CX3CL1-binding protein 2

Supplemental Table ST5. Top 5% of Identity-by-descent (IBD) regions in 2019-2020 Mali isolates from Ouélessébougou.

Chr	Start	End	Fraction	Location	Gene ID	Gene product	Gene name
6	1110001	1120000	0.02074037	1114544-1117537	PF3D7_0627800	acetyl-CoA synthetase, putative	ACS
6	1200001	1210000	0.02281915	1205190-1207781	PF3D7_0629300	phospholipase, putative	PL
6	1210001	1220000	0.02544293	1210420-1212762	PF3D7_0629400	polyadenylate-binding protein 3, putative	PABP3
6	1210001	1220000	0.02544293	1213948-1216005	PF3D7_0629500	amino acid transporter AAT1	AAT1
6	1220001	1230000	0.02996695	1221941-1242922	PF3D7_0629700	SET domain protein, putative	SET1
6	1230001	1240000	0.03621427	1221941-1242922	PF3D7_0629700	SET domain protein, putative	SET1
6	1240001	1250000	0.04015512	1221941-1242922	PF3D7_0629700	SET domain protein, putative	SET1
6	1250001	1260000	0.04146341	1254907-1256940	PF3D7_0630100	alpha/beta hydrolase, putative	/
7	370001	380000	0.0249511	372897-373106	PF3D7_0708100	DNA-directed RNA polymerases I, II, and III subunit RPABC5, putative	RPB10
7	380001	390000	0.03311512	385583-388321	PF3D7_0708500	heat shock protein 86 family protein	/
7	390001	400000	0.04070598	395711-398332	PF3D7_0708800	heat shock protein 110	HSP110c
7	390001	400000	0.04070598	391502-392188	PF3D7_0708700	Cg8 protein	/
7	400001	410000	0.0493389	403222-406317	PF3D7_0709000	chloroquine resistance transporter	CRT
7	420001	430000	0.05203866	428723-429346	PF3D7_0709500	nucleic acid-binding protein, putative	/
7	430001	440000	0.04008451	435089-436195	PF3D7_0709700	prodrug activation and resistance esterase	PARE
7	460001	470000	0.01624402	463105-471837	PF3D7_0710200	conserved Plasmodium protein, unknown function	/
13	100001	110000	0.01553341	99548-100521	PF3D7_1301700	CX3CL1-binding protein 2	CBP2

SUPPLEMENTAL FILES

Supplemental File 1
Samples included in the analysis (SupplementalFile1.xlsx)
