# Supplementary material for: Genome-wide genetic variation and molecular surveillance of drug resistance in Plasmodium falciparum isolates from asymptomatic individuals in Ouélessébougou, Mali
Source: Sci Rep. 2023 Jun 12;13:9522. doi: 10.1038/s41598-023-36002-w (PMC10258750; doi:10.1038/s41598-023-36002-w)
Supplement: Supplementary file 3 — Supplementary Information 3. [file 41598_2023_36002_MOESM3_ESM.pdf]

Variation génétique à l'échelle du génome et surveillance moléculaire de la résistance aux médicaments dans les isolats de *Plasmodium falciparum* provenant des personnes asymptomatiques à Ouélessébougou, au Mali.

Leen N. Vanheer[1], Almahamoudou Mahamar[2], Emilia Manko[1], Sidi M. Niambele[2], Koualy Sanogo[2], Ahamadou Youssouf[2], Adama Dembele[2], Makonon Diallo[2], Seydina O. Maguiraga[2], Jody Phelan[1], Ashley Osborne[1], Anton Spadar[1], Merel J. Smit[3], Teun Bousema[3], Chris Drakeley[1], Taane G. Clark[1,4], William Stone[1], Alassane Dicko[2], Susana Campino[1]

[1] Faculty of Infectious and Tropical Diseases, London School of Hygiene & Tropical Medicine, London, UK

[2] Malaria Research and Training Centre, Faculty of Pharmacy and Faculty of Medicine and Dentistry, University of Sciences Techniques and Technologies of Bamako, Bamako, Mali

[3] Department of Medical Microbiology and Radboud Center for Infectious Diseases, Radboud University Medical Center, Nijmegen, Netherlands

[4] Faculty of Epidemiology and Population Health, London School of Hygiene & Tropical Medicine, London, UK

Auteurs correspondants

Dr. Leen Vanheer et Prof. Susana Campino

Department of Infection Biology

London School of Hygiene & Tropical Medicine,

London WC1E 7HT, UK

leen.vanheer@lshtm.ac.uk, susana.campino@lshtm.ac.uk

Résumé

L'analyse de la séquence des parasites *Plasmodium falciparum* est utile pour garantir le succès durable des programmes de lutte contre le paludisme. Les technologies de séquençage du génome entier donnent un aperçu de l'épidémiologie et de la variation à l'échelle du génome des populations de *P. falciparum* et peuvent caractériser les changements géographiques et temporels. Cela est particulièrement important pour surveiller l'émergence et la propagation de parasites de *P. falciparum* résistants aux médicaments, qui menacent les programmes de lutte contre le paludisme dans le monde entier. Nous présentons ici une caractérisation détaillée de la variation génétique à l'échelle du génome et des profils de résistance aux médicaments chez des personnes asymptomatiques dans le sud-ouest du Mali, où la transmission du paludisme est intense et saisonnière, et où le nombre de cas a récemment augmenté. Des échantillons collectés à Ouélessébougou, au Mali (2019-2020 ; n=87) ont été séquencés et placés dans le contexte d'isolats de *P. falciparum* maliens (2007-2017 ; n=876) et africains (n=711) plus anciens. Notre analyse a révélé une forte multiclonalité et une faible parenté entre les isolats, ainsi que des fréquences accrues de marqueurs moléculaires de résistance à la sulfadoxine-pyriméthamine et à la luméfantrine, par rapport aux isolats maliens plus anciens. En outre, 21 gènes soumis à une pression sélective ont été identifiés, dont un candidat vaccin bloquant la transmission (*pfCelTOS*) et un locus d'invasion érythrocytaire (*pfdblmsp2)*. Dans l'ensemble, notre travail fournit l'évaluation la plus récente de la diversité génétique de *P. falciparum* au Mali, un pays où le paludisme occupe la deuxième place en Afrique de l'Ouest, ce qui permet d'éclairer les activités de lutte contre le paludisme.

INTRODUCTION

On estime que le paludisme sera à l'origine de 250 millions de maladies dans le monde et de 619 000 décès associés rien qu'en 2021. Le Mali fait partie des 9 pays où la charge de morbidité du paludisme est la plus élevée et le nombre de cas de paludisme a augmenté entre 2016 et 2021 [1]. La plupart des cas de paludisme au Mali et dans le reste de l'Afrique subsaharienne sont causés par *Plasmodium falciparum,* le parasite le plus virulent du paludisme humain. Le Mali est divisé en cinq zones écoclimatiques [2] à travers lesquelles la transmission du paludisme fluctue. La zone du Sud-Ouest a les taux d'incidence de *P. falciparum les* plus élevés et la transmission est fortement saisonnière, coïncidant avec la saison des pluies annuelle. Cependant, dans les différentes régions du sud-ouest du Mali, le degré de transmission pérenne et saisonnière et varie, tout comme le moment de la saison des pluies, ce qui entraîne une hétérogénéité des saisons de transmission et de l'épidémiologie du paludisme [2].

Conformément aux directives de l'OMS, les stratégies actuelles d'intervention contre le paludisme au Mali comprennent des combinaisons thérapeutiques à base d'artémisinine (CTA) pour le paludisme simple à *P. falciparum*, l'artéméther-luméfantrine (AL) étant le traitement de première intention. Les stratégies de chimioprévention du paludisme saisonnier (CPS), consistant en l'association de sulfadoxine-pyriméthamine et d'amodiaquine (SPAQ) chez les enfants et le traitement préventif intermittent des femmes enceintes (IPTp) avec la sulfadoxine-pyriméthamine (SP) seulement, ont été largement mises en œuvre dans les pays africains et ont été introduites au Mali à partir de 2012 et de 2015, respectivement.

Outre les perturbations dues à la pandémie de COVID-19, l'augmentation de l'incidence de la maladie a été liée à l'émergence et à la propagation de parasites *P. falciparum* résistants aux médicaments [1]. Les souches résistantes à la SP, aux dérivés de l'artémisinine et aux médicaments partenaires constituent un défi majeur dans la lutte contre le paludisme [3]. Les marqueurs moléculaires de la résistance aux médicaments sont donc extrêmement utiles pour identifier et surveiller les parasites *P. falciparum*

résistants aux médicaments et ont été décrits pour la plupart des médicaments antipaludiques. Des mutations dans le gène de résistance aux médicaments multiples (*pfmdr1*), par exemple, ont été associées à diverses réponses des parasites à la luméfantrine, à la chloroquine (CQ), à l'amodiaquine, à la méfloquine et à la pipéraquine. La base génétique de la résistance à la SP est bien documentée et implique une accumulation de mutations dans le gène de la dihydrofolate-réductase (*pfdhfr*) (N51I, C59R, S108N et I164L) et dans le gène de la dihydroptéroate-synthase (*pfdhps*) (S436A/F, A437G, K540E, A581G et A613S/T). Les infections hébergeant le triple mutant *dhfr* C**IRN**I (mutations soulignées) sont courantes dans toute l'Afrique et sont résistantes à la pyriméthamine [4], [5]. [4], [5]. La combinaison de ce triple mutant *dhfr* avec le double mutant dhps (A437G et K540E, S**GE**AA) augmente encore le risque d'échec du traitement par SP à 50 % [5], [6].

Des mutations du gène *pfkelch13* associées à une sensibilité réduite à l'artémisinine sont apparues et se sont répandues en Asie du Sud-Est et sont apparues plus récemment dans plusieurs pays d'Afrique de l'Est [7]-[9]. On s'attend à ce que ce phénomène s'étende à d'autres régions d'Afrique et il est donc essentiel de surveiller en permanence les variations génétiques de *pfkelch13*. La forte prévalence de la résistance à la CQ a conduit à son retrait de toutes les directives de traitement des infections à *P. falciparum* en Afrique subsaharienne. Des décennies plus tard, des parasites *P. falciparum* sensibles à la CQ sont réapparus dans de nombreuses régions du monde [10]-[13]. C'est pourquoi il a été proposé de réintroduire la CQ, en association avec d'autres antipaludiques [14]. Les rapports du Mali n'ont pas observé de diminution substantielle de la fréquence des mutations du transporteur de la résistance à la chloroquine (*pfcrt*) associées à la résistance à la CQ [15], bien qu'une tendance à la baisse ait été récemment signalée dans des isolats maliens collectés en 2016-2017 [16]. Il est donc important de poursuivre l'évaluation afin de déterminer si la CQ pourrait être réintroduite dans la région à l'avenir.

La majorité des infections paludéennes dans les zones endémiques sont asymptomatiques [17]. Cependant, ces porteurs ont tendance à être sous-représentés dans les analyses génomiques à grande échelle, en raison à la fois de l'absence de recherche de traitement et des difficultés techniques liées au séquençage des infections à faible densité. Les porteurs asymptomatiques sont les principaux

contributeurs au réservoir infectieux, car ils peuvent rester infectieux pendant de longues périodes sans présenter de symptômes, ce qui signifie qu'ils peuvent transmettre la maladie à d'autres sans savoir qu'ils sont infectés. En outre, leur fréquence dans la population et les caractéristiques des individus qui sont plus susceptibles d'être asymptomatiques, comme un risque plus élevé de piqûres de moustiques, augmentent encore leur contribution au réservoir infectieux [18], [19]. Il est donc essentiel de comprendre les caractéristiques génétiques du réservoir asymptomatique pour contrôler efficacement la propagation du paludisme.

Les progrès des technologies de séquençage de nouvelle génération (NGS) ont rendu le séquençage du génome entier (WGS) plus accessible et plus abordable pour la gestion des maladies et la lutte contre le paludisme. Outre l'identification et le suivi des marqueurs moléculaires de la résistance aux médicaments, l'étude des variations génomiques est utile pour comprendre la dynamique de la transmission, les balayages sélectifs et l'épidémiologie de *P. falciparum. L'*évaluation des changements dans la parenté génomique au sein d'une population, y compris en utilisant des mesures d'identité par descendance, peut donner des indications sur la démographie de la population parasitaire et l'intensité de la transmission au fil du temps [20]. En outre, l'identification des gènes soumis à une sélection peut donner des indications sur la pression sélective exercée par les médicaments ou d'autres agents inconnus, ce qui est important pour élaborer des stratégies efficaces de prévention, de contrôle et de traitement du paludisme.

En résumé, la surveillance de la résistance aux médicaments de *P. falciparum* est essentielle pour informer les politiques pharmaceutiques dans le monde entier, en particulier dans les régions à forte transmission du paludisme, et l'OMS recommande une mise à jour et une surveillance régulière de la résistance aux médicaments antipaludiques pour soutenir les progrès accomplis dans la lutte contre le paludisme et son élimination. [21] . Dans ce rapport, nous fournissons une analyse approfondie des profils de résistance aux médicaments et des variations génomiques récentes, en utilisant la sélection, l'ascendance et l'analyse de l'identité par descendance, chez les personnes asymptomatiques à Ouélessébougou et dans les villages voisins, au Mali, en 2019 et 2020. En outre, nous les plaçons dans

le contexte des isolats maliens de *P. falciparum* collectés entre 2007 et 2017, ainsi que des populations parasitaires à l'échelle de l'Afrique.

RÉSULTATS

Structure génomique des populations maliennes de P. falciparum

L'analyse SNP à l'échelle du génome de 962 isolats maliens de *P. falciparum,* collectés entre 2007 et 2020 dans deux études différentes et provenant de 9 endroits (**figure 1A**), a révélé des différences dans la multiplicité de l'infection et la structure de la population. Les isolats de Ouélessébougou collectés en 2019-2020 provenaient d'infections asymptomatiques à *P. falciparum,* tandis qu'aucun détail sur la présentation clinique n'était disponible pour les génomes disponibles publiquement dans la base de données MalariaGEN. Les taux d'incidence de *P. falciparum* ont varié d'un site d'échantillonnage à l'autre et sur deux décennies (**figure 1A-B**). Au total, 863 046 SNP de haute qualité ont été identifiés. La multiclonalité a été mesurée à l'aide de la métrique F_{ws} , ou coefficient de consanguinité, qui indique une monoclonalité si elle est > 0,95, tandis qu'une métrique F_{ws} plus faible indique une multiclonalité. La valeur moyenne de F_{ws} pour les échantillons du Mali 2019-2020 (n=87) provenant de Ouélessébougou était de 0,80, avec seulement 20% des échantillons abritant un seul clone. Nous avons constaté une multiclonalité élevée dans les isolats de Ouélessébougou 2019-2020 par rapport aux isolats provenant de différents sites où la collecte a eu lieu entre 2015 et 2017 (**figure 1C**). En utilisant les données SNP, une analyse statistique UMAP (Uniform Manifold Approximation and Projection) pour regrouper les isolats, a révélé peu de sous-structure spatiale entre les populations, bien qu'un certain regroupement basé sur le lieu (**Figure 1D**) et l'année de collecte (**Figure 1E**) puisse être observé et que les isolats de Ouélessébougou forment un sous-groupe individuel.

Structure génomique des populations maliennes de P. falciparum par rapport aux populations africaines

Une visualisation UMAP basée sur les SNP et un arbre du maximum de vraisemblance montrent comment les isolats de Ouélessébougou les plus récemment collectés sont apparentés aux populations africaines (n=875) et ont révélé une similarité avec les isolats d'Afrique de l'Ouest et d'Afrique centrale

(**figure 2A-B**). Aucun groupe distinct ne peut être détecté, ce qui indique une forte parenté et suggère des mouvements d'informations génétiques au sein de ces populations, qui peuvent se produire à la fois par le biais de la migration humaine et de la migration des vecteurs. Les isolats d'Afrique de l'Est, d'Afrique du Sud-Est et d'Afrique centrale forment des groupes distincts, tandis que la Corne de l'Afrique semble constituer un groupe distinct au sein de la population d'Afrique de l'Est. L'évaluation de la multiclonalité montre que les isolats maliens de 2019-2020 sont relativement plus multiclonaux que les isolats collectés dans d'autres pays d'Afrique de l'Ouest (**figure 2C**).

Fréquences des marqueurs moléculaires de résistance aux médicaments

Des fréquences accrues des marqueurs moléculaires de la sulfadoxine-pyriméthamine et la persistance des marqueurs de résistance à la chloroquine ont été observées dans la population de Ouélessébougou 2019-2020 (n=87), par rapport aux isolats maliens de 2007-2017 (n=876) (**Tableau ST1**). Les polymorphismes provoquant des changements d'acides aminés qui confèrent une résistance à la chloroquine dans le *pfcrt* (K76T, A220S, Q271E, N326S, I356T et R371I) ont persisté à des fréquences similaires au fil du temps (**Figure 3A-B**). Le polymorphisme N86Y sur *pfmdr1* a diminué au fil du temps, passant de 28,4 % en 2007 à 2,6 % en 2020. Cet allèle 86Y a été associé à la résistance à la chloroquine et à l'amodiaquine ainsi qu'à la résistance à la pipéraquine (allèle 86Y en combinaison avec Y184F), tandis que les parasites porteurs de l'allèle N86 sont moins sensibles à la luméfantrine, à la pipéraquine et à la méfloquine [22], [23]. Les modifications des acides aminés Y184F et D1246Y dans le MDR1 ont persisté à des fréquences comparables. Trois SNP non synonymes dans *pfmdr1* qui n'avaient pas été trouvés auparavant dans les isolats maliens, entraînant des changements d'acides aminés S400C, D431Y et K503N, ont été identifiés dans 2,13% des isolats de 2019 et 2,94% des isolats de 2020, respectivement (**tableau ST1**). La fréquence des mutations ponctuelles du *pfdhfr* associées à la résistance à la pyriméthamine (N51I, C59R et S108N) a approximativement doublé depuis 2007, atteignant des fréquences alarmantes de 92,4 %, 93,9 % et 92,7 %, respectivement, en 2020. En outre, l'haplotype C**IRN**I triple mutant a augmenté en fréquence et représentait 82,7 % de la population de parasites en 2019-2020, tandis que l'haplotype de type sauvage était réduit à 4,9 % (**figure 3C, tableau ST2**). Une mutation du *pfdhps* conférant une résistance à la sulfadoxine (A437G) a vu sa fréquence

augmenter de 27,7 % en 2007 à 74,3 % en 2020, tandis que S436A a montré une tendance à la baisse (**figure 3D**). La mutation *pfdhps* K540E a été trouvée dans 4 isolats en 2019-2020 et toutes ces mutations ont été combinées avec le triple mutant *dhfr du* C**IRN**I, conduisant à une fréquence de mutant quadruple de 2,47%. Un SNP non synonyme en position 748145 dans *pfdhfr* (V20I), a été nouvellement identifié dans les isolats maliens en 2019, à une fréquence de 2,04%. Aucune mutation connue de *pfkelch13* associée à la résistance à l'artémisinine n'a été identifiée dans les isolats maliens. Les mutations R255K, K189N et K189T de *pfkelch13* ont persisté à des fréquences similaires à celles observées en 2007 et aucune nouvelle mutation de *pfkelch13* n'a été identifiée (**tableau ST1**).

Régions soumises à une pression sélective dans les isolats maliens

La détermination des régions génomiques soumises à une sélection directionnelle par l'analyse de la structure des haplotypes dans les isolats de Ouélessébougou 2019-2020 et en comparaison avec les populations maliennes plus anciennes, a révélé qu'un certain nombre de gènes étaient soumis à une pression sélective. Le score d'haplotype intégré (*iHS)* a été utilisé pour identifier les SNP sous sélection dans la population 2019-2020 (**Figure 4A)** et les régions du génome avec un nombre élevé de SNP sous sélection (**Tableau ST3**). Cela a permis d'identifier des gènes codant pour des protéines conservées de *Plasmodium* dont la fonction est inconnue (PF3D7_0212100 et PF3D7_0425100), principalement exprimés dans les ookinètes et les stades annulaires, respectivement, ainsi que le gène *pfCelTOS*, qui code pour une protéine de traversée des cellules pour les ookinètes et les sporozoïtes et qui a été suggéré comme un antigène candidat intéressant pour les vaccins [24], [25]. L'indice *Rsb* entre les populations a été utilisé pour identifier les SNP en cours de sélection lors de la comparaison de la population 2019-2020 avec les isolats maliens plus anciens collectés en 2007-2014 (n=414) (**Figure 4B**) et 2015-2017 (n=462) (**Figure 4C).** Les régions présentant un nombre élevé de SNP sous sélection ont également été déterminées **(tableau ST4**). Cela a permis d'identifier des gènes associés à l'invasion des érythrocytes (*pfdblmsp2, pfmsp3*), au transport des protéines (*pfMC-2TM*), à la cytoadhérence (*pfCLAG3.2*), ainsi qu'un gène codant pour un ARN de fonction inconnue (Pf3D7_0421400, RUF6).

L'analyse des mélanges ancestraux confirme que les isolats maliens ont des ancêtres similaires

L'estimation de l'ascendance spatiale des isolats maliens et des populations africaines a révélé des origines ancestrales similaires pour tous les isolats maliens de *P. falciparum*. Le nombre optimal de populations ancestrales a été estimé à 5 (K=5 ; K1-K5), sur la base de la décroissance des valeurs propres correspondant à K allant de 1 à 10. La population ancestrale K1 était dominante dans les échantillons gambiens (49,4 %), tandis que la population ancestrale K2 semblait être liée aux populations du Centre-Sud et de l'Afrique de l'Est (Malawi, 92,3 % ; Madagascar, 74,4 % ; Tanzanie, 69 % ; Kenya, 61,9 %). Les isolats maliens, ainsi que les isolats mauritaniens, semblaient contenir principalement la population ancestrale K3 (Mali, 80,3 % ; Mauritanie, 80,5 %), ainsi que de plus petites portions d'ascendance K2 (Mali, 8,5 % ; Mauritanie, 7,7 %) et K4 (Mali, 10 % ; Mauritanie, 10,1 %). De très faibles fractions d'ascendance K1 et K5 étaient présentes dans les isolats maliens et mauritaniens, à l'exception des isolats de Bandiagara au Mali qui ne semblaient pas contenir d'ascendance K5 (**Figure 5**).

L'analyse de l'identité par descendance révèle la grande diversité de la population de Ouélességougou

En tant que mesure de la parenté génétique au sein des populations, l'analyse de l'identité par descendance (IBD) a révélé que les isolats de Ouélességougou présentent de très faibles fractions d'IBD par paire à travers le génome (médiane = 0, intervalle = 0 - 0,133), tandis que les isolats maliens collectés en 2007-2014 et 2015-2017 ont montré une parenté légèrement plus élevée (médiane = 0,021 et médiane = 0,017, respectivement) (**Figure S2**). Les 5 % de positions IBD les plus élevées (classées dans des fenêtres de 10 kb du génome) dans les isolats de Ouélességougou étaient réparties dans 17 régions sur 3 chromosomes (chr. 6, 7 et 13) (**Figure S3**, **Tableau ST5**). Trois régions à forte IBD sur le chromosome 6 incluaient le gène codant pour l'histone méthyltransférase SET1 et le *gène pfcrt* sur le chromosome 7 a également été identifié comme englobant une forte IBD.

DISCUSSION

Les technologies NGS ont fourni une méthode de plus en plus réalisable pour explorer la variation génomique à l'échelle du génome et la dynamique des populations de parasites du paludisme. Ici, nous avons fourni une analyse détaillée de la diversité génomique des isolats de *P. falciparum* provenant de porteurs de gamétocytes asymptomatiques dans 11 villages de Ouélessébougou en 2019 et 2020 et les avons placés dans le contexte d'isolats maliens précédemment séquencés, ainsi que d'isolats à l'échelle africaine via une analyse SNP à l'échelle du génome. Nous avons constaté une forte multiclonalité et une faible parenté entre les isolats et identifié des gènes soumis à une pression sélective. Nous avons également observé des fréquences accrues de marqueurs moléculaires de résistance à la sulfadoxine-pyriméthamine et à la luméfantrine, par rapport aux isolats maliens plus anciens.

Les génomes des isolats maliens de *P. falciparum* collectés entre 2007 et 2017 ont été générés précédemment [16], [26]. Cependant, une stratégie d'échantillonnage plus actualisée et plus systématique est nécessaire pour soutenir les efforts de contrôle et d'élimination de l'infection. . Ouélessébougou est une communauté rurale de la région de Koulikoro, dans le sud-ouest du Mali, qui présente l'un des taux d'incidence de *P. falciparum les* plus élevés du pays [27]. Idéalement, dans de tels contextes, il devrait y avoir un suivi régulier des changements dans la variation génomique, afin de déterminer les schémas épidémiologiques et la dynamique de la population, en particulier parmi les porteurs asymptomatiques de gamétocytes, et d'évaluer l'impact des mesures de contrôle de l'infection. Par exemple, une surveillance continue des marqueurs moléculaires de la résistance aux médicaments est nécessaire pour informer les approches de la chimiothérapie du paludisme.

Les isolats maliens présentent un regroupement mineur en utilisant la visualisation UMAP, les isolats de Ouélessébougou formant un sous-groupe. Sur un arbre de maximum de vraisemblance, les isolats de Ouélessébougou et d'autres isolats maliens se regroupent avec les populations d'Afrique de l'Ouest, comme prévu. Malgré un rapport récent observant une diminution de la multiplicité de l'infection de 2007 à 2017 au Mali [16] , nos résultats montrent une multiclonalité plus élevée en 2019-2020 que dans

les isolats provenant de différents sites où la collecte a eu lieu entre 2015 et 2017. Le statut hautement multiclonal de la population parasitaire étudiée peut s'expliquer par de multiples facteurs, tels que les taux d'incidence élevés de *P. falciparum* dans cette région (Fig. 1B), le moment de la collecte des échantillons pendant la saison de transmission maximale (septembre-décembre) et la présentation clinique asymptomatique des individus. Comme les porteurs asymptomatiques de gamétocytes de *P. falciparum* sont moins susceptibles de chercher un traitement, ces infections peuvent se prolonger, ce qui augmente la probabilité de réinfection et de multiclonalité. Bien que la plupart des données de séquençage disponibles proviennent d'infections incidentes, il est important de caractériser génétiquement les porteurs asymptomatiques de *P. falciparum, car ce* sont eux qui contribuent le plus au réservoir infectieux et qui sont à l'origine des infections qui échappent au traitement [18].

Les stratégies de traitement du paludisme dans le monde ont été modifiées au fil du temps en raison de l'émergence de parasites résistants aux anciens médicaments de première intention, dans le but de préserver l'efficacité des médicaments antipaludiques et de réduire le fardeau mondial du paludisme. Nous avons constaté que les fréquences des marqueurs moléculaires conférant une résistance à la CQ ont persisté dans les isolats maliens 2019-2020 à des fréquences similaires à celles d'il y a dix ans, malgré son retrait de toutes les directives de traitement de *P. falciparum* depuis 2006 et un rapport faisant état d'une tendance à la baisse en 2017 au Mali [16]. Cette situation est différente de celle d'autres régions du monde où un retour de la sensibilité à la CQ a été observé [11]-[14], [28] et pourrait indiquer un faible coût d'adaptation associé au maintien des polymorphismes de résistance au *pfcrt* dans la population ou une utilisation continue de la CQ en vente libre. [29]-[31], soulignant ainsi la nécessité d'étudier et de réduire la disponibilité de ce médicament au Mali.

Nous avons observé une augmentation de la variante N86 *pfmdr1* de 71,6 % en 2007 à 97,4 % en 2020. Cet allèle N86 a été associé à une moindre sensibilité à la luméfantrine, à la pipéraquine et à la méfloquine [22], [23] , tandis que l'allèle 86Y a précédemment montré une résistance à la chloroquine et à l'amodiaquine, ainsi qu'à la pipéraquine. 86Y a précédemment montré une résistance à la chloroquine et à l'amodiaquine, ainsi qu'une résistance à la pipéraquine (allèle 86Y en combinaison

avec Y184F). Par conséquent, il a été constaté que l'AL sélectionnait l'allèle N86, tandis que l'artésunate-amodiaquine et la dihydroartémisinine-pipéraquine sélectionnent l'allèle 86Y. [32]-[35] . Le génotypage N86Y pourrait donc être un marqueur utile pour guider la rotation des CTA dans une zone géographique donnée [36]. Ainsi, l'augmentation observée de l'allèle N86 peut refléter une proportion croissante d'isolats présentant une sensibilité réduite à l'AL, ce qui soulève des inquiétudes quant à l'utilisation continue de l'AL en tant que CTA de première ligne au Mali. Une faible fréquence de l'allèle 86Y a déjà été rapportée au Mali en 2016, ainsi que dans d'autres pays africains où l’AL est largement utilisée [15], [34]. Ce résultat contraste avec l'augmentation de la fréquence de l'allèle 86Y observée précédemment chez les enfants de Ouélessébougou entre 2014 et 2016, après 3 ans de CPS avec SPAQ, qui était probablement due à la sélection de l'allèle 86Y par l'amodiaquine [37]. Cependant, il est important de noter que, bien qu'une diminution de la sensibilité à l’AL des isolats porteurs de l'allèle N86 ait été observée dans des isolats provenant de plusieurs pays africains [22], [32], [33] .

Des fréquences élevées de mutants C**IRN**I triple *pfdhfr* (82,72 %) ont été observées dans les isolats de Ouélessébougou de 2019-2020, ce qui représente une augmentation substantielle par rapport aux années précédentes (34,3 %, 55,1 % et 70,7 % en 2007, 2013-2014 et 2015-2017, respectivement). Le mutant *pfdhps* K540E, qui était quasi absent dans les isolats maliens collectés entre 2007 et 2017, a été retrouvé dans 5,9% des isolats de 2019 et 2,7% des isolats de 2020. En outre, tous les isolats mutants K540E portaient le triple mutant *dhfr. Une* surveillance continue est donc nécessaire pour contrôler les niveaux de résistance à la SP et l'émergence de mutants quadruple et quintuple (en combinaison avec *dhps* A437G ou *dhps* A581G). En outre, les cartes de prévalence des mutations en ligne telles que wwarn.org SP molecular surveyor deviendront de plus en plus utiles pour évaluer le médicament à utiliser pour la CPS [38]. Aucun SNP à des positions du gène *pfkelch13* qui ont été signalées comme entraînant une diminution de la sensibilité aux dérivés de l'artémisinine n'a été observé dans les isolats maliens. Cependant, il est nécessaire de poursuivre la surveillance des mutants *pfkelch13* au cours des prochaines années, car on peut s'attendre à une propagation de ces allèles à partir de l'Afrique de l'Est ou à leur émergence indépendante.

L'analyse de la structure des haplotypes dans les isolats de Ouélessébougou de 2019-2020 a identifié des SNP soumis à une pression sélective dans le gène *pfCelTOS*, qui est impliqué dans la motilité de glissement des sporozoïtes, la traversée des cellules, et qui est un candidat vaccinal bloquant la transmission [24], [25]. Une pression sélective a été observée à cet emplacement génomique avant le début de l'épidémie de la grippe aviaire. La pression sélective a déjà été observée à cet emplacement génomique [39], ce qui indique un degré élevé de diversité et fait de *pfCelTOS* un candidat-vaccin moins intéressant. Une analyse croisée des populations comparant les populations de parasites de 2019-2020 à celles de 2007-2014 et 2015-2017 a été réalisée et, malgré l'augmentation des fréquences des marqueurs moléculaires de la résistance aux médicaments au cours de cette période de 13 ans, nous n'avons trouvé aucun SNP associé à la résistance aux médicaments soumis à une pression sélective directionnelle. Nous avons identifié des SNP soumis à une pression sélective dans des gènes associés à l'invasion des érythrocytes (*pfdblmsp2)* dans les deux comparaisons de populations et des gènes associés au transport des protéines (*pfMC-2TM*), à la cytoadhérence (*pfCLAG3.2*) et à un gène codant pour un ARN de fonction inconnue (Pf3D7_0421400, RUF6) dans la comparaison entre les populations de 2019-2020 et de 2015-2017. La pression sélective dans *pfdblmsp2* et les membres de la famille multigénique clag a déjà été décrite et est probablement due à leur emplacement en tant que protéines de surface et au contact avec le système immunitaire de l'hôte qui en résulte [39], [40]. L'analyse IBD a révélé de très faibles fractions d'IBD par paire à travers le génome dans les isolats de Ouélessébougou 2019-2020, ce qui indique une faible parenté entre les isolats. Le gène *pfcrt*, associé à la résistance à la CQ et à la pipéraquine, se trouvait dans les 5 % de positions IBD les plus élevées, ce qui suggère que ce gène est très conservé parmi les isolats de Ouélessébougou. Ceci est en accord avec les fréquences persistantes observées des marqueurs moléculaires de la résistance à la CQ. En outre, le gène *pfSET1*, une histone lysine méthyltransférase d'importation, était également très conservé. L'analyse de l'ascendance du mélange a montré des ascendances similaires pour tous les isolats maliens, ce qui est en accord avec un rapport récent [16].

Cette étude présente plusieurs limites. Tout d'abord, un biais d'échantillonnage ne peut être exclu car nous n'avons séquencé que des parasites provenant d'infections asymptomatiques et d'individus âgés de

5 à 50 ans, alors que les ensembles de données accessibles au public ne précisent pas la présentation clinique ou l'âge de l'individu. En outre, l'analyse du nombre de copies de la *plasmepsine 1-2*, qui confère la résistance à la pipéraquine, n'a pas été réalisée, en raison de l'amplification sélective du génome entier (sWGA) de l'ADN du parasite avant le séquençage, qui empêche une analyse adéquate du nombre de copies. Enfin, nous n'avons pas évalué la résistance phénotypique ou toute association entre le génotype et le phénotype, mais les marqueurs moléculaires de la résistance aux médicaments rapportés ici ont été largement prouvés pour prédire les niveaux de résistance aux médicaments *in vitro* ou les résultats du traitement des patients [4]-[6], [41]-[43].

Après des années de progrès dans la réduction de la charge mondiale du paludisme, les taux d'incidence et les décès sont maintenant en hausse. Cette augmentation pourrait être due aux perturbations des programmes de lutte contre le paludisme pendant la pandémie de COVID-19, mais la résistance aux antipaludiques a également été liée à cette augmentation. Pour progresser vers l'objectif de réduire de 90 % le fardeau du paludisme dans le monde d'ici à 2030 [44], il est nécessaire de mettre en place des programmes de lutte contre le paludisme. Nous devons obtenir une image complète de la variation génomique et de l'épidémiologie des populations de parasites. Ici, nous avons fourni une évaluation actualisée de la diversité génomique de la population de parasites *P. falciparum* dans le sud-ouest du Mali, une région où la transmission du paludisme est très intense. Nos résultats montrent que les parasites originaires du Mali sont regroupés en fonction de leur origine géographique et temporelle, les isolats de Ouélessébougou formant un sous-groupe distinct. Ceci suggère une grande diversité génétique parmi les isolats maliens. Les marqueurs moléculaires de la résistance à la SP sont en augmentation, ce qui montre une progression vers l'échec de cette association médicamenteuse et nécessite une surveillance continue. Aucune diminution de la résistance à la CQ n'a été observée au fil du temps, ce qui s'oppose à l'idée d'une réintroduction potentielle de la CQ au Mali dans un avenir proche. Notre étude fournit des données précieuses sur le scénario épidémiologique actuel et la résistance aux médicaments du paludisme au Mali et peut contribuer à un contrôle efficace du paludisme au Mali. D'autres applications des approches de séquençage, y compris les nouvelles technologies

portables et les tests de séquençage d'amplicons, dans les pays où le paludisme est endémique sont nécessaires pour aider au contrôle de la maladie et informer les directives de traitement.

MÉTHODES

Sites d'étude

En 2019 et 2020, un total de 180 personnes présentant des gamétocytes de *P. falciparum* détectables au microscope en l'absence de symptômes de paludisme ont été recrutées dans le cadre de deux essais cliniques [45], [46] à Ouélessébougou et dans 11 villages de Ouélessébougou, au Mali (**Figure S1**). Ouélessébougou est une commune qui comprend la ville de Ouélessébougou et 44 villages environnants, soit un total d'environ 50 000 habitants. La ville est située à environ 80 km au sud de Bamako, la capitale du Mali. La transmission du paludisme à Ouélessébougou est très saisonnière et se produit pendant la saison des pluies, de juillet à novembre. Les données WGS publiquement disponibles sur les isolats maliens proviennent de huit autres sites du sud du Mali, essentiellement des villages ruraux (Bougoula-Hameau, Dangassa, Faladje, Kenieroba, Kolle, Nioro-du-Sahel, Bandiagara) et d'une zone urbaine (Bamako). Tous les sites ont un climat subtropical avec des saisons sèches et pluvieuses, à l'exception de Nioro-du-Sahel, qui est caractérisé par un climat désertique [2], [16].

Collecte d'échantillons et séquençage du génome entier

Au total, 97 échantillons de sang total ont été prélevés sur des porteurs de gamétocytes de *P. falciparum*, âgés de 5 à 50 ans, recrutés dans le cadre de deux essais cliniques précédemment publiés à Ouélessébougou [45], [46]. L'autorisation de mener cette étude a été obtenue auprès du Comité d'éthique de la recherche de la London School of Hygiene and Tropical Medicine (numéros de référence 17507 et 21905) et du Comité d'éthique de l'Université des Sciences Techniques et Technologies de Bamako (numéros de référence 2019/67/CE/FMPOS et 2020/96/CE/FMPOS/FAPH) et a été réalisée conformément aux directives et réglementations en vigueur. Les essais ont été enregistrés sur ClinicalTrials.gov (NCT04049916 et NCT04609098). Le consentement éclairé écrit de tous les sujets et/ou de leurs tuteurs légaux a été obtenu avant le prélèvement des échantillons. Pour les participants

mineurs, le consentement éclairé pour la participation à l'étude a été obtenu de leurs parents et/ou de leur tuteur légal. L'identification des espèces a été effectuée au microscope par des microscopistes formés au Centre de recherche et de formation sur le paludisme de l'Université de Bamako (Bamako, Mali). L'ADN a été extrait de 83,3 µL de sang total à l'aide d'un extracteur automatisé MagNAPure LC (Total Nucleic Acid Isolation Kit High Performance ; Roche Applied Science, Indianapolis, IN, USA) et amplifié à l'aide d'un ensemble d'amorces et d'un protocole d'amplification sélective du génome entier (sWGA) établis. [47], [48]. Le séquençage du génome entier a été réalisé sur une plateforme Illumina Novaseq 6000 chez Eurofins Genomics, en Allemagne, ce qui a permis d'obtenir un minimum de 3,75 millions de lectures appariées (lectures de 250 pb) par échantillon.

Sélection des ensembles de données, cartographie des lectures, détection des variantes et contrôle de la qualité

Au total, 1701 isolats de *P. falciparum* ont été inclus dans l'analyse (**fichier supplémentaire S1)**, y compris des séquences de génomes entiers accessibles au public Malaria Genetic Epidemiology Network [26] (MalariaGEN) (Pf Community Project, n = 1141 ; SPOTmalaria project, n = 463) et des isolats nouvellement séquencés (n = 97) provenant de personnes asymptomatiques infectées par *P. falciparum* et recrutées dans le cadre de deux essais cliniques précédemment publiés à Ouélessébougou, au Mali [45], [46]. Toutes les données brutes ont été filtrées à l'aide de *trimmomatic* (version 0.39) et des paramètres suivants : LEADING:3 TRAILING:3 SLIDINGWINDOW:4:20 MINLEN:36. Les lectures Illumina ont ensuite été mises en correspondance avec le génome de référence de *P. falciparum* (Pf3D7 ; v3) à l'aide du logiciel *bwa-mem* (v0.7.17). Les SNP et les insertions et délétions courtes ont été appelés à l'aide des logiciels *samtools* (v1.12) et GATK (v4.1.4.1). Les SNP à appel mixte ont été assignés à des génotypes déterminés par un ratio de couverture dans lequel les appels de nucléotides étaient de 80 % ou plus. Les échantillons présentant plus de 40 % de lacunes n'ont été inclus dans aucune analyse. Sur les 97 échantillons nouvellement séquencés, 9 ont été retirés en raison de l'absence de réponse et un a été retiré après que la prédiction de l'espèce a identifié *P. malariae* (https://github.com/jodyphelan/malaria-profiler), laissant un total de 87 (89,6 %) isolats nouvellement séquencés dans les analyses finales. Parmi les ensembles de données accessibles au public, 8 ont été

supprimés, ce qui a donné un total de 1673 échantillons inclus dans les analyses, y compris des isolats du Cameroun (n = 99), de la République démocratique du Congo (RDC ; n = 98), de la Gambie (n = 80), du Kenya (n = 91), du Malawi (n = 97), du Mali (n = 962), de la Mauritanie (n = 79), de la Tanzanie (n = 120), de Madagascar (n = 22) et de l'Éthiopie (n = 25).

Analyses génétiques des populations

La visualisation de la géographie des sites d'échantillonnage a été réalisée à l'aide des logiciels R *ggmap* (version 3.0.0) et *tmap* (version 3.3.3). Les taux d'incidence de *P. falciparum* pour la période 2000-2020 ont été consultés sur le site du Malaria Atlas Project [27] . Les graphiques UMAP (Uniform Manifold Approximation and Projection) ont été créés à l'aide du package R *uwot* avec la métrique "hamming" et les paramètres par défaut. Un arbre de vraisemblance maximale a été créé en appliquant le logiciel *iqtree* en utilisant les SNP du génome entier, et la visualisation a été effectuée dans *iTOL* (version 6*)* [49], [50]. Pour les analyses génétiques des populations qui comparent les isolats maliens aux populations africaines, un sous-ensemble d'isolats maliens collectés entre 2007 et 2017 a été utilisé (y compris 10 isolats de chaque site et de chaque année de collecte, si disponible), afin d'obtenir un nombre comparable d'isolats entre les populations. La multiclonalité a été déterminée en calculant la métrique F_{ws}, à l'aide d'un script interne qui utilise le package R moimix (https://github.com/bahlolab/moimix) pour évaluer la diversité intra-hôte par rapport à la diversité de la population locale. Seuls les SNP bi-alléliques dans les régions codantes ont été utilisés pour les calculs et un filtrage des fréquences des allèles mineurs (MAF) de 0,1 % a été effectué afin d'inclure exclusivement les SNP robustes. Les MAF dans les gènes de résistance aux médicaments ont été extraits de la matrice binaire et annotés à l'aide du logiciel *CSQ de Bcftools*, qui identifie la mutation comme non synonyme, synonyme ou intergénique, ainsi que le décalage de codon et de protéine causé par toute mutation non synonyme [51]. Seules les positions génomiques avec des MAF d'au moins 2 % ont été retenues dans l'analyse. La visualisation des données a été réalisée à l'aide du progiciel R *ggplot2* (R version 4.1.2). Les régions du génome soumises à une sélection directionnelle ont été identifiées à l'aide du progiciel R *rehh* (version 3.2.2), qui utilise des mesures basées sur la population de la diversité des haplotypes au sein des populations (*iHS)* ou entre les populations (*Rsb*) [52]. Le progiciel R *Tess3r*

(version 1.1.0, utilisant les paramètres par défaut à l'exception de "rep=25") a été utilisé pour calculer le mélange sur la base de la modélisation spatiale du partage des allèles en utilisant les coordonnées géographiques des sites d'échantillonnage en plus des données SNP pangénomiques [53]. Le nombre optimal d'ascendances a été déterminé pour différents nombres de sous-populations (K = 1, 2, ..., 10). L'analyse IBD pour les isolats avec F_{ws} > 0,85 a été réalisée pour évaluer la connectivité entre les parasites au sein des populations. Pour ce faire, on a estimé la fraction d'ascendance partagée par paire entre les segments génomiques, dont on a déduit qu'ils descendaient d'un ancêtre commun récent. Ces fractions IBD ont été calculées à l'aide du logiciel *hmmIBD* avec les paramètres par défaut, qui déploie une approche basée sur un modèle de Markov caché [54].

Remerciements

Nous souhaitons remercier les participants à l'étude et le personnel du centre de santé communautaire de Ouélessébougou et des villages environnants pour leur coopération, ainsi que le moniteur de sécurité local et les membres du comité de surveillance des données et de la sécurité pour leur supervision. Nous remercions le centre de ressources MalariaGEN pour la génération des données génétiques publiées.

Déclaration de financement

Cette analyse a été réalisée sur des génomes séquencés à partir d'échantillons de sang collectés dans le cadre de deux essais cliniques au Mali, financés par la Fondation Bill & Melinda Gates (#INV-002098). LNV bénéficie d'une bourse de doctorat du Biotechnology and Biological Sciences Research Council (BBSRC) LSHTM-tethered PhD award. SC est financé par le Medical Research Council UK (subvention n° MR/M01360X/1). TGC bénéficie du soutien du Medical Research Council UK (subventions n° MR/M01360X/1, MR/N010469/1, MR/R020973/1, MR/X005895/1). Les financeurs n'ont joué aucun rôle dans la conception de l'étude, la collecte et l'analyse des données, la décision de publier ou la préparation du manuscrit.

Contributions des auteurs

A Dicko, WS, CD, MJS, TB et SC ont conçu et élaboré l'étude ; AM, SMN, KS, AY, A Dembele, MD, SO et A Dicko ont coordonné et effectué le recrutement et la collecte des échantillons ; LNV a traité les échantillons pour le séquençage. LNV, SC et CD ont coordonné le séquençage des échantillons ; LNV, EM, JP, AO et AS ont effectué l'analyse bioinformatique et statistique, sous la supervision de SC et TGC ; LNV a rédigé la première version du manuscrit, et la version finale a été révisée par tous les auteurs. Le manuscrit final a été lu et approuvé par tous les auteurs.

Intérêts concurrents

Les auteurs ne déclarent aucun intérêt concurrent.

Disponibilité des données

Les ensembles de données présentés dans cette étude se trouvent dans les Archives européennes de nucléotides (ENA). Les noms et les numéros d'accès des échantillons figurent dans le fichier supplémentaire S1. Les séquences brutes des isolats séquencés dans cette étude sont disponibles sur le site web de l'ENA (Project accession PRJEB60381).

RÉFÉRENCES

[1] Organisation mondiale de la santé, *Rapport sur le paludisme dans le monde 2022*. Genève : Organisation mondiale de la santé, 2022. Consulté : 18 déc. 2022. [En ligne]. Disponible : https://www.who.int/teams/global-malaria-programme/reports/world-malaria-report-2022

[2] M. Cissoko *et al*, 'Geo-Epidemiology of Malaria at the Health Area Level, Dire Health District, Mali, 2013-2017', *Int. J. Environ. Res. Public. Health*, vol. 17, no. 11, p. 3982, Jun. 2020, doi : 10.3390/ijerph17113982.

[3] M. Dhorda, C. Amaratunga, et A. M. Dondorp, 'Artemisinin and multidrug-resistant Plasmodium falciparum - a threat for malaria control and elimination', *Curr. Opin. Infect. Dis.*, vol. 34, no. 5, p. 432, Oct. 2021, doi : 10.1097/QCO.0000000000000766.

[4] W. Sirawaraporn, T. Sathitkul, R. Sirawaraporn, Y. Yuthavong et D. V. Santi, "Antifolate-resistant mutants of Plasmodium falciparum dihydrofolate reductase", *Proc. Natl. Acad. Sci. U. S. A.*, vol. 94, no. 4, pp. 1124-1129, Feb. 1997, doi : 10.1073/pnas.94.4.1124.

[5] S. G . Staedke, H. Sendagire, S. Lamola, M. R. Kamya, G. Dorsey et P. J. Rosenthal, 'Relationship between age, molecular markers, and response to sulphadoxine-pyrimethamine treatment in Kampala, Uganda', *Trop. Med. Int. Health*, vol. 9, no. 5, pp. 624-629, 2004, doi : 10.1111/j.1365-3156.2004.01239.x.

[6] J. G. Kublin *et al*, 'Molecular Markers for Failure of Sulfadoxine-Pyrimethamine and Chlorproguanil-Dapsone Treatment of Plasmodium falciparum Malaria', *J. Infect. Dis.*, vol. 185, no. 3, pp. 380-388, Feb. 2002, doi : 10.1086/338566.

[7] A. Uwimana *et al*, 'Emergence and clonal expansion of in vitro artemisinin-resistant Plasmodium falciparum kelch13 R561H mutant parasites in Rwanda', *Nat. Med.*, vol. 26, no. 10, pp. 1602-1608, Oct. 2020, doi : 10.1038/s41591-020-1005-2.

[8] B. Balikagala *et al*, 'Evidence of Artemisinin-Resistant Malaria in Africa', *N. Engl. J. Med.*, vol. 385, no. 13, pp. 1163-1171, Sep. 2021, doi : 10.1056/NEJMoa2101746.

[9] B. H. Stokes *et al*, 'Plasmodium falciparum K13 mutations in Africa and Asia impact artemisinin resistance and parasite fitness', *eLife*, vol. 10, p. e66277, Jul. 2021, doi : 10.7554/eLife.66277.

[10] A. E. P. Frosch *et al*, 'Return of Widespread Chloroquine-Sensitive Plasmodium falciparum to Malawi', *J. Infect. Dis.*, vol. 210, no. 7, pp. 1110-1114, oct. 2014, doi : 10.1093/infdis/jiu216.

[11] K. K . Asare, J. Africa, J. Mbata, and Y. K. Opoku, 'The emergence of chloroquine-sensitive Plasmodium falciparum is influenced by selected communities in some parts of the Central Region of Ghana', *Malar. J.*, vol. 20, no. 1, p. 447, Nov. 2021, doi : 10.1186/s12936-021-03985-8.

[12] O. Dagnogo *et al*, " Vers une réémergence de la sensibilité à la chloroquine en Côte d'Ivoire ? ", *Malar. J.*, vol. 17, no. 1, p. 413, nov. 2018, doi : 10.1186/s12936-018-2551-7.

[13] B. J. Njiro, R. F. Mutagonda, A. T. Chamani, T. Mwakyandile, D. Sabas, and G. M. Bwire, 'Molecular surveillance of chloroquine-resistant Plasmodium falciparum in sub-Saharan African countries after withdrawal of chloroquine for treatment of uncomplicated malaria : A systematic review', *J. Infect. Public Health*, vol. 15, no. 5, pp. 550-557, mai 2022, doi : 10.1016/j.jiph.2022.03.015.

[14] J. G. Kublin *et al*, 'Reemergence of chloroquine-sensitive Plasmodium falciparum malaria after cessation of chloroquine use in Malawi', *J. Infect. Dis.*, vol. 187, no. 12, pp. 1870-1875, Jun. 2003, doi : 10.1086/375419.

[15] S. A. S. Diakité *et al*, 'A comprehensive analysis of drug resistance molecular markers and Plasmodium falciparum genetic diversity in two malaria endemic sites in Mali', *Malar. J.*, vol. 18, no. 1, p. 361, nov. 2019, doi : 10.1186/s12936-019-2986-5.

[16] A. Coulibaly *et al*, 'Genome-wide SNP analysis of Plasmodium falciparum shows differentiation at drug-resistance-associated loci among malaria transmission settings in southern Mali', *Front. Genet*, vol. 13, p. 943445, Oct. 2022, doi : 10.3389/fgene.2022.943445.

[17] T. Bousema, L. Okell, I. Felger, and C. Drakeley, 'Asymptomatic malaria infections : detectability, transmissibility and public health relevance', *Nat. Rev. Microbiol*, vol. 12, no. 12, pp. 833-840, déc. 2014, doi : 10.1038/nrmicro3364.

[18] F. G. Tadesse *et al*, "The Relative Contribution of Symptomatic and Asymptomatic Plasmodium vivax and Plasmodium falciparum Infections to the Infectious Reservoir in a Low-Endemic Setting in Ethiopia" (La contribution relative des infections symptomatiques et asymptomatiques à Plasmodium vivax et Plasmodium falciparum au réservoir infectieux dans un contexte de faible endémicité en Éthiopie), *Clin. Infect. Dis. Off. Publ. Infect. Dis. Soc. Am.*, vol. 66, no. 12, pp. 1883-1891, Jun. 2018, doi : 10.1093/cid/cix1123.

[19] B. P. Gonçalves *et al*, 'Examining the human infectious reservoir for Plasmodium falciparum malaria in areas of differing transmission intensity', *Nat. Commun*, vol. 8, no. 1, p. 1133, déc. 2017, doi : 10.1038/s41467-017-01270-4.

[20] D. E. Neafsey, A. R. Taylor et B. L. MacInnis, "Advances and opportunities in malaria population genomics", *Nat. Rev. Genet*, vol. 22, no. 8, Art. no. 8, août 2021, doi : 10.1038/s41576-021-00349-5.

[21] E. Patouillard, J. Griffin, S. Bhatt, A. Ghani et R. Cibulskis, " Global investment targets for malaria control and elimination between 2016 and 2030 ", *BMJ Glob. Health*, vol. 2, no. 2, p. e000176, mai 2017, doi : 10.1136/bmjgh-2016-000176.

[22] M. Malmberg *et al*, 'Plasmodium falciparum drug resistance phenotype as assessed by patient antimalarial drug levels and its association with pfmdr1 polymorphisms', *J. Infect. Dis.*, vol. 207, no. 5, pp. 842-847, mars 2013, doi : 10.1093/infdis/jis747.

[23] M. I. Veiga *et al*, 'Globally prevalent PfMDR1 mutations modulate Plasmodium falciparum susceptibility to artemisinin-based combination therapies', *Nat. Commun.*, vol. 7, no. 1, p. 11553, mai 2016, doi : 10.1038/ncomms11553.

[24] E. S. Bergmann-Leitner, P. M. Legler, T. Savranskaya, C. F. Ockenhouse, et E. Angov, 'Cellular and humoral immune effector mechanisms required for sterile protection against sporozoite challenge induced with the novel malaria vaccine candidate CelTOS', *Vaccine*, vol. 29, no. 35, pp. 5940-5949, août 2011, doi : 10.1016/j.vaccine.2011.06.053.

[25] E. S. Bergmann-Leitner *et al*, 'Self-adjuvanting bacterial vectors expressing pre-erythrocytic antigens induce sterile protection against malaria', *Front. Immunol.*, vol. 4, p. 176, 2013, doi : 10.3389/fimmu.2013.00176.

[26] MalariaGEN *et al*, 'Pf7 : an open dataset of Plasmodium falciparum genome variation in 20,000 worldwide samples', *Wellcome Open Res*, vol. 8, p. 22, Jan. 2023, doi : 10.12688/wellcomeopenres.18681.1.

[27] D. A. Pfeffer *et al*, 'malariaAtlas : an R interface to global malariometric data hosted by the Malaria Atlas Project', *Malar. J.*, vol. 17, no. 1, p. 352, oct. 2018, doi : 10.1186/s12936-018-2500-5.

[28] H. Ocholla *et al*, 'Whole-Genome Scans Provide Evidence of Adaptive Evolution in Malawian Plasmodium falciparum Isolates', *J. Infect. Dis.*, vol. 210, no. 12, pp. 1991-2000, Dec. 2014, doi : 10.1093/infdis/jiu349.

[29] A. E. Frosch, M. Venkatesan, et M. K. Laufer, 'Patterns of chloroquine use and resistance in sub-Saharan Africa : a systematic review of household survey and

molecular data', *Malar. J.*, vol. 10, no. 1, p. 116, mai 2011, doi : 10.1186/1475-2875-10-116.

[30] A. Paloyo et A. Reichert, "Biting Back at Malaria : Assessing Health-service Providers' Compliance with Treatment Guidelines", *Rev. Dev. Econ.*, vol. 21, no. 3, pp. 591-626, 2017, doi : 10.1111/rode.12283.

[31] Office des Nations Unies contre la drogue et le crime, *Trafic de produits médicaux au Sahel*. Nations Unies, 2023. doi : 10.18356/9789210025409.

[32] S. D. Otienoburu *et al*, 'Selection of Plasmodium falciparum pfcrt and pfmdr1 polymorphisms after treatment with artesunate-amodiaquine fixed dose combination or artemether-lumefantrine in Liberia', *Malar. J.*, vol. 15, no. 1, p. 452, Sep. 2016, doi : 10.1186/s12936-016-1503-3.

[33] C. Sisowath *et al*, 'In vivo selection of Plasmodium falciparum pfmdr1 86N coding alleles by artemether-lumefantrine (Coartem)', *J. Infect. Dis.*, vol. 191, no. 6, pp. 1014-1017, mars 2005, doi : 10.1086/427997.

[34] A. R. Taylor *et al*, 'Artemether-Lumefantrine and Dihydroartemisinin-Piperaquine Exert Inverse Selective Pressure on Plasmodium Falciparum Drug Sensitivity-Associated Haplotypes in Uganda', *Open Forum Infect. Dis.*, vol. 4, no. 1, p. ofw229, 2017, doi : 10.1093/ofid/ofw229.

[35] P. Sondo *et al*, 'Artesunate-Amodiaquine and Artemether-Lumefantrine Therapies and Selection of Pfcrt and Pfmdr1 Alleles in Nanoro, Burkina Faso', *PLoS ONE*, vol. 11, no. 3, p. e0151565, Mar. 2016, doi : 10.1371/journal.pone.0151565.

[36] J. P. Gil et S. Krishna, " pfmdr1 (Plasmodium falciparum multidrug drug resistance gene 1) : a pivotal factor in malaria resistance to artemisinin combination therapies ", *Expert Rev. Anti Infect. Ther.*, vol. 15, no. 6, pp. 527-543, Jun. 2017, doi : 10.1080/14787210.2017.1313703.

[37] A. Mahamar *et al*, 'Effect of three years' seasonal malaria chemoprevention on molecular markers of resistance of Plasmodium falciparum to sulfadoxine-pyrimethamine and amodiaquine in Ouelessebougou, Mali', *Malar. J.*, vol. 21, no. 1, p. 39, Feb. 2022, doi : 10.1186/s12936-022-04059-z.

[38] Observatoire des données sur les maladies infectieuses (IDDO), 'SP Molecular Surveyor', *Worldwide Antimalarial Resistance Network*, 02 février 2015. https://www.wwarn.org/tracking-resistance/sp-molecular-surveyor (consulté le 10 mai 2023).

[39] A. Osborne *et al*, 'Characterizing the genomic variation and population dynamics of Plasmodium falciparum malaria parasites in and around Lake Victoria, Kenya', *Sci. Rep*, vol. 11, no. 1, p. 19809, Oct. 2021, doi : 10.1038/s41598-021-99192-1.

[40] D. Abera, C. K. Kibet, T. Degefa, L. Amenga-Etego, J. L. Bargul, and L. Golassa, 'Genomic analysis reveals independent evolution of Plasmodium falciparum populations in Ethiopia', *Malar. J.*, vol. 20, no. 1, p. 129, mars 2021, doi : 10.1186/s12936-021-03660-y.

[41] H. A. Babiker, S. J. Pringle, A. Abdel-Muhsin, M. Mackinnon, P. Hunt, et D. Walliker, 'High-Level Chloroquine Resistance in Sudanese Isolates of *Plasmodium falciparum* Is Associated with Mutations in the Chloroquine Resistance Transporter Gene *pfcrt* and the Multidrug Resistance Gene *pfmdr1*', *J. Infect. Dis.*, vol. 183, no. 10, pp. 1535-1538, mai 2001, doi : 10.1086/320195.

[42] G. Mombo-Ngoma *et al*, 'High prevalence of dhfr triple mutant and correlation with high rates of sulphadoxine-pyrimethamine treatment failures in vivo in Gabonese children', *Malar. J.*, vol. 10, p. 123, mai 2011, doi : 10.1186/1475-2875-10-123.

[43] A. F. Cowman, M. J. Morry, B. A. Biggs, G. A. Cross et S. J. Foote, "Amino acid changes linked to pyrimethamine resistance in the dihydrofolate reductase-thymidylate

synthase gene of Plasmodium falciparum", *Proc. Natl. Acad. Sci. U. S. A.*, vol. 85, no. 23, pp. 9109-9113, Dec. 1988, doi : 10.1073/pnas.85.23.9109.

[44] Organisation mondiale de la santé, *Stratégie technique mondiale de lutte contre le paludisme 2016-2030*. Genève : Organisation mondiale de la santé, 2015. Consulté : 12 janvier 2023. [En ligne]. Disponible : https://apps.who.int/iris/handle/10665/176712

[45] W. Stone *et al*, 'Single low-dose tafenoquine combined with dihydroartemisinin-piperaquine to reduce Plasmodium falciparum transmission in Ouelessebougou, Mali : a phase 2, single-blind, randomised clinical trial', *Lancet Microbe*, vol. 0, no. 0, Mar. 2022, doi : 10.1016/S2666-5247(21)00356-6.

[46] W. Stone *et al*, Pyronaridine-artesunate or dihydroartemisinin-piperaquine combined with single low-dose primaquine to prevent Plasmodium falciparum malaria transmission in Ouélessébougou, Mali : a four-arm, single-blind, phase 2/3, randomised trial', *Lancet Microbe*, vol. 3, no. 1, pp. e41-e51, Jan. 2022, doi : 10.1016/S2666-5247(21)00192-0.

[47] S. O. Oyola *et al*, 'Whole genome sequencing of Plasmodium falciparum from dried blood spots using selective whole genome amplification', *Malar. J.*, vol. 15, no. 1, p. 597, déc. 2016, doi : 10.1186/s12936-016-1641-7.

[48] E. L. Clarke, S. A. Sundararaman, S. N. Seifert, F. D. Bushman, B. H. Hahn et D. Brisson, "swga : a primer design toolkit for selective whole genome amplification", *Bioinforma. Oxf. Engl.*, vol. 33, no. 14, pp. 2071-2077, juil. 2017, doi : 10.1093/bioinformatics/btx118.

[49] I. Letunic and P. Bork, 'Interactive Tree Of Life (iTOL) : an online tool for phylogenetic tree display and annotation', *Bioinformatics*, vol. 23, no. 1, pp. 127-128, Jan. 2007, doi : 10.1093/bioinformatics/btl529.

[50] B. Q. Minh *et al*, 'IQ-TREE 2 : New Models and Efficient Methods for Phylogenetic Inference in the Genomic Era', *Mol. Biol. Evol*, vol. 37, no. 5, pp. 1530-1534, mai 2020, doi : 10.1093/molbev/msaa015.

[51] P. Danecek et S. A. McCarthy, " BCFtools/csq : haplotype-aware variant consequences ", *Bioinformatics*, vol. 33, no. 13, p. 2037-2039, juil. 2017, doi : 10.1093/bioinformatics/btx100.

[52] M. Gautier, A. Klassmann, et R. Vitalis, 'rehh 2.0 : a reimplementation of the R package rehh to detect positive selection from haplotype structure', *Mol. Ecol. Resour*, vol. 17, no. 1, pp. 78-90, 2017, doi : 10.1111/1755-0998.12634.

[53] K. Caye, T. M. Deist, H. Martins, O. Michel, et O. François, 'TESS3 : fast inference of spatial population structure and genome scans for selection', *Mol. Ecol. Resour.*, vol. 16, no. 2, pp. 540-548, 2016, doi : 10.1111/1755-0998.12471.

[54] S. F. Schaffner, A. R. Taylor, W. Wong, D. F. Wirth, and D. E. Neafsey, 'hmmIBD : software to infer pairwise identity by descent between haploid genotypes', *Malar. J.*, vol. 17, no. 1, p. 196, mai 2018, doi : 10.1186/s12936-018-2349-7.

FIGURE LEGENDS

Figure 1. Structure de la population des isolats de *P. falciparum* collectés au Mali de 2007 à 2020. (A) Carte du Mali présentant le nombre de cas de *Plasmodium falciparum* nouvellement diagnostiqués pour 1 000 habitants en 2020 (échelle de couleurs) et indiquant les sites de collecte des études MalariaGEN (cercle) et des essais cliniques New Drug Combinations for *P. falciparum* Transmission Reduction (NECTAR) (triangle), colorés par le nom du village ou de la ville. Cette carte a été générée à l'aide du package R *tmap* (version 3.3.3 ; https://r-tmap.github.io/tmap/) **(B)** Taux d'incidence de *P. falciparum* pour chaque site de collecte (par couleur) de 2000 à 2020. **(C)** Complexité des infections estimée par le coefficient de reproduction (métrique F_{ws}) par échantillon, classé par site d'étude, la couleur indiquant l'année de collecte. La ligne de tendance grise en pointillés est à 0,95, les lignes en pointillés indiquent la valeur moyenne de F_{ws} par groupe. **(D, E)** Visualisation de 962 isolats maliens de *P. falciparum par* approximation et projection du manifold uniforme (UMAP), colorés par site et par année de collecte, respectivement.

Figure 2. Structure de la population des isolats maliens de *P. falciparum dans le* contexte des populations africaines. (A) Visualisation de 962 isolats de *P. falciparum* à l'échelle de l'Afrique, dont 87 isolats collectés au Mali en 2019-2020 et 164 isolats maliens collectés en 2007-2020, par approximation et projection uniformes des plis (UMAP). Les régions africaines où les isolats ont été collectés sont indiquées en couleur. **(B)** Arbre du maximum de vraisemblance du même ensemble de données, annoté par les isolats maliens de 2019-2020 (anneau intérieur), la région africaine d'origine de l'échantillon (deuxième anneau), l'année de collecte (troisième anneau) et le pays ouest-africain de collecte (anneau extérieur). **(C)** Complexité des infections (coefficient F_{ws}) par échantillon pour chaque échantillon, groupé par pays et séparant les isolats maliens 2019-2020 des isolats maliens 2007-2017. La ligne de tendance grise en pointillés est à 0,95, les lignes en pointillés indiquent la valeur moyenne de F_{ws} par groupe.

Figure 3. Prévalence des polymorphismes nucléotidiques simples connus pour entraîner une diminution de la sensibilité aux médicaments. Les fréquences des allèles mineurs (MAF) sont indiquées pour les isolats collectés de 2007 à 2020 au Mali pour les gènes associés à la résistance aux médicaments, y compris **(A)** *pfcrt*, **(B)** *pfmdr1*, **(C)** *pfdhfr* et **(D)** *pfdhps*. Les diagrammes circulaires en C et D représentent les fréquences auxquelles des combinaisons de mutants *pfhdfr* et *pfdhps* ont été observées dans les isolats de *P. falciparum* collectés en 2007, 2013-2014, 2015-2017 et 2019-2020.

Figure 4. Recherche de preuves d'une sélection directionnelle récente. Les diagrammes de Manhattan montrent l'analyse du **(A)** score d'haplotypes intégré (*iHS)* pour les SNP individuels dans la population du Mali 2019-2020 et le test *Rsb* inter-populations pour les haplotypes étendus comparant la population du Mali 2019-2020 à la **(B)** population du Mali 2007-2014 et à la **(C)** population du Mali 2015-2017.

Figure 5. Proportions d'ascendance de mélange à l'échelle du génome pour les populations de *P. falciparum* sur l'ensemble du continent africain. (A) Ascendances par isolat (colonnes) pour chaque pays, classées par année de collecte croissante. L'astérisque indique les isolats de Bandiagara au Mali. **(B)** Carte géographique des ascendances estimées en utilisant K=5 populations ancestrales sur le continent africain.
